# Low‐intensity pulsed ultrasound improves myocardial ischaemia‒reperfusion injury via migrasome‐mediated mitocytosis

**DOI:** 10.1002/ctm2.1749

**Published:** 2024-07-01

**Authors:** Ping Sun, Yifei Li, Weidong Yu, Jianfeng Chen, Pingping Wan, Zhuo Wang, Maomao Zhang, Chao Wang, Shuai Fu, Ge Mang, Stephen Choi, Zhuo Du, Caiying Tang, Song Li, Guoxia Shi, Jiawei Tian, Jiannan Dai, Xiaoping Leng

**Affiliations:** ^1^ Department of Ultrasound The Second Affiliated Hospital of Harbin Medical University Harbin China; ^2^ Ultrasound Molecular Imaging Joint Laboratory of Heilongjiang Province Harbin China; ^3^ The Key Laboratory of Myocardial Ischemia Harbin Medical University, Ministry of Education Harbin China; ^4^ Laboratory of Animal Center The Second Affiliated Hospital of Harbin Medical University Harbin China; ^5^ Department of Cardiology The Second Affiliated Hospital of Harbin Medical University Harbin China; ^6^ SXULTRASONIC Ltd. Kerry Rehabilitation Medicine Research Institute Shenzhen China

**Keywords:** low‐intensity pulsed ultrasound, mitochondria, mitocytosis, myocardial ischaemia‒reperfusion injury

## Abstract

During myocardial ischaemia‒reperfusion injury (MIRI), the accumulation of damaged mitochondria could pose serious threats to the heart. The migrasomes, newly discovered mitocytosis‐mediating organelles, selectively remove damaged mitochondria to provide mitochondrial quality control. Here, we utilised low‐intensity pulsed ultrasound (LIPUS) on MIRI mice model and demonstrated that LIPUS reduced the infarcted area and improved cardiac dysfunction. Additionally, we found that LIPUS alleviated MIRI‐induced mitochondrial dysfunction. We provided new evidence that LIPUS mechanical stimulation facilitated damaged mitochondrial excretion via migrasome‐dependent mitocytosis. Inhibition the formation of migrasomes abolished the protective effect of LIPUS on MIRI. Mechanistically, LIPUS induced the formation of migrasomes by evoking the RhoA/Myosin II/F‐actin pathway. Meanwhile, F‐actin activated YAP nuclear translocation to transcriptionally activate the mitochondrial motor protein KIF5B and Drp1, which are indispensable for LIPUS‐induced mitocytosis. These results revealed that LIPUS activates mitocytosis, a migrasome‐dependent mitochondrial quality control mechanism, to protect against MIRI, underlining LIPUS as a safe and potentially non‐invasive treatment for MIRI.

## Graphical abstract

LIPUS serves as a promising non‐pharmacological and non‐invasive therapeutic strategy to ameliorate MIRI.

LIPUS induces the formation of migrasomes by triggering the RhoA/Myosin II pathway and mediates mitocytosis through the Myosin II/YAP/Drp1/KIF5B motor protein axes.

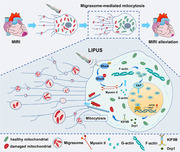

## 1 INTRODUCTION

Acute myocardial infarction (AMI), the most severe form of coronary heart disease, has a high fatality rate, affecting an average of more than 7 million people each year.^1^, ^2^ Existing treatment strategies mainly focus on thrombolysis or revascularisation, which inevitably cause grievous and irreversible myocardial ischaemic/reperfusion injury (MIRI), leading to further damage and accelerating cardiomyocyte death.^3^ Mitochondrial injury is considered to be a pivotal trigger responsible for the pathological progress of MIRI.^4^, ^5^, ^6^ MIRI can lead to mitochondrial damage. In turn, as damaged mitochondria accumulate, a large number of inflammasomes are activated, striking cardiac metabolism and steady‐state transformation, and sequentially increasing the burden and detriment to the heart.^7^, ^8^

Notably, mitochondria undergo dynamic changes during the process of mitochondrial injury. The delivery and discharge of mitochondria are effective methods for restoring damaged cardiomyocytes.^8^ Migrasomes are newly discovered membrane organelles that depend on cell displacement.^9^ When cells migrate, retraction fibres drawn from the cells ‘back‐end’ of the cells break, leaving migrasomes behind. It has been reported that migrasomes manage crucial cellular processes, including cell‒cell communication, lateral mRNA and protein transfer, and mitochondrial quality control.^10^, ^11^, ^12^ They can package signal molecules and be engulfed by surrounding cells, providing a mechanism for material transfer between cells for cellular communications. Migrasomes can also remain on the migration path of the migrating cell, acting as a positional signal after the cell has left. Intracellular contents and unwanted materials can be released and shed through them. Similarly, impaired mitochondria are packaged and selectively removed in a process called mitocytosis to maintain homeostasis.^12^ Therefore, driving migrasome‐mediated mitocytosis to enhance mitochondrial quality control may be a promising strategy for MIRI therapy.

Recently, low‐intensity pulsed ultrasound (LIPUS) has been shown to be an effective low‐cost biophysical therapy for the treatment of cardiovascular diseases. LIPUS could reduce the infarct size in AMI, repair damaged myocardium, alleviate adverse left ventricular remodelling and improve cardiac function.^13^, ^14^ Nevertheless, at present, the therapeutic effects of LIPUS and its underlying mechanisms need to be further elucidated.

LIPUS could induce elastic vibration, increase cell activity and fluid‐medium movement, and further promote mass transfer and reaction velocity.^15^ Of note, through this mechanical stimulation, LIPUS can modulate mitochondrial dynamic changes and mitochondrial quality control. RhoA, a molecule highly sensitive to mechanical stimuli, could further drive cytoskeletal rearrangement to promote cell migration, beginning with the establishment of cell polarity and the formation of prominence in the movement direction.^16^, ^17^, ^18^, ^19^, ^20^ Meanwhile, RhoA could activate Myosin II, which is a key molecule in migrasome formation.^21^, ^22^, ^23^ RhoA activation exerts cardio‐ and mitochondrial protection against MIRI.^24^ However, whether LIPUS forces cells to activate RhoA to drive migrasome‐mediated mitocytosis to protect MIRI is unclear.

In this study, we demonstrated that LIPUS ameliorated MIRI through migrasome‐mediated mitocytosis to facilitate the elimination of damaged mitochondria via a RhoA‐dependent pathway. Our findings may provide a novel, non‐invasive and non‐drug solution for the treatment of MIRI.

## 2 METHODS

### 2.1 Experimental MIRI mouse model

Male C57BL/6J mice (6−8 weeks old, 18–20 g) were purchased from the Beijing Vital River Laboratory Animal Technology. All animals were housed and cared for in a specific pathogen‐free facility, strictly adhering to the Guide for the Care and Use of Laboratory Animals (Institute of Laboratory Animal Resources). All animal experiments and protocols were approved by the Committee on the Ethics of Animal Experiments of the Second Affiliated Hospital of Harbin Medical University (approval no. sydwgzr2021‐139).

Mice were anaesthetised and placed in a static cage for 30 min prior to surgery, with half of the cage placed on a warm water blanket set at 38°C for prewarming. After anesthesia with an intraperitoneal injection of 30 mg/kg sodium pentobarbital, the animals were intubated and ventilated with a rodent ventilator. A parasternal thoracotomy was performed between the third and fourth ribs. The left anterior descending (LAD) artery was visualised and ligated using a 6‐0 silk suture. All animals underwent LAD coronary artery occlusion for 30 min, followed by 2 h of reperfusion or a sham operation. To recover from anesthesia, the mice were placed on a warm blanket set at 38°C before being returned to the social housing.

Mouse heart tissue was harvested after terminal anaesthesia (1.2% isoflurane inhalation) and euthanasia by cervical dislocation at indicated time. An anesthetic vaporiser fed by a supply of a gas mixture containing 21% oxygen balanced with nitrogen was used to deliver isoflurane in the gas phase until a concentration of 1%−2% isoflurane was reached, and the atmosphere was supplemented with 5% carbon dioxide.

### 2.2 LIPUS treatment

A low‐frequency ultrasound probe was attached to the chest of the mice, and an ultrasound coupling agent was applied. The LIPUS device (SXULTRASONIC) was then connected, and the following parameters were set: centre frequency of 1 MHz, pulse repetition frequency of 1000 Hz, pressure intensity of 0.25 W/cm^2^ and duty ratio of 20%. The LIPUS group received treatment for 20 min three times a day on days 0−7, and twice a week during weeks 2−4 under inhalation anaesthesia with 1.2% isoflurane. In contrast, the LIPUS‐free group received placebo treatment.

For cellular LIPUS treatment, AC16, H9C2, HUVECs cell lines and neonatal mouse cardiomyocytes (NMCMs) were cultured and treated with I/R. Then, the LIPUS was applied to the cultured cells through an agar phantom gel with a 1.0‐MHz centre frequency, 20% duty cycle, the pulse repetition frequency of 1000 Hz and indicated pressure intensity. The LIPUS group was given LIPUS treatment for 20 min once, whereas the control group underwent the same procedures without the LIPUS therapy.

### 2.3 Generation of endothelial cell‐specific TSPAN4 knockout mice

The mice with tamoxifen‐inducible Cre‐fusion protein under the control of the endothelial cell‐specific Cdh5 promoter (Cdh5‐CreERT2) were crossed with TSPAN4 ^flox/flox^ mice. TSPAN4^flox/flox^ mice were generated by TSPAN4 ^flox/‒^ mice on C57BL/6J background. Cdh5‐CreERT2and TSPAN4^flox/−^ mice were purchased from Cyagen Biosciences Inc. Inducible deletion of TSPAN4 in the Cdh5‐CreERT2 model was achieved by tamoxifen treatment (40 mg/kg for three consecutive days by i.p. injection). Cre negative Flox littermates were used as controls.

### 2.4 Generation of cardiomyocyte‐specific TSPAN4 knockout mice

The TSPAN4^flox/flox^ mice were hybridised with αMHC‐MerCreMer transgenic mice (Cyagen Biosciences Inc.) to deplete TSPAN4 expression in adult cardiomyocytes under tamoxifen administration. For TSPAN4‐CKO (TSPAN4^flox/flox^ crossed with αMHC‐MerCreMer) mice, tamoxifen was administered at the dose of 40 mg/kg i.p. for three consecutive days. Cre negative Flox littermates were used as controls.

### 2.5 Confocal imaging for visualisation of migrasomes and mitocytosis

For plasmid transfection and live‐cell imaging, lipofectamine (Lipo) 3000 was used according to the manufacturer's instructions. Cells were seeded in a confocal dish precoated with fibronectin (10 mg/mL) at a density of 8 × 10^3^ and grown to 60%−70% confluency, and then transfected with OptiMEM which contained 0.4 µL Lipo 3000, 0.25 µg TSPAN4‐GFP (GenePharma) and 0.25 µg MitoDsRed (Fenghui Biology). The transfection mix system was replaced with fresh medium after 4 h, and the cells were cultured for another 12–24 h for confocal microscopy.

To capture migrasome‐mediated mitocytosis, cells stained with wheat germ agglutinin (WGA) were immersed in Hank's balanced salt solution(HBSS) for 10 min and MitoSOX or MitoRed in HBSS for 15 min. Confocal images were acquired using a ZEISS microscope (Oberkochen).

### 2.6 Statistical analyses

For all experiments, the results are expressed as mean ± standard deviation. Comparisons of variables among groups were performed using Student's *t*‐test or one‐way analysis of variance, followed by Tukey's post hoc tests when appropriate. Data were analysed using GraphPad Prism 9.0 (GraphPad Software). Statistical significance was defined as a *p*‐value < 0.05.

## 3 RESULTS

### 3.1 LIPUS improves cell viability and survival post‐I/R

We used I/R‐treated AC16 cell lines to evaluate the efficacy of LIPUS on cell viability. We observed that when the magnitude of pressure conditions varied from 0.2 to 0.25, 0.3, 0.4 and 0.5 W/cm^2^, LIPUS treatment significantly increased cell viability, with a maximum effect noted at 0.25 W/cm^2^ (Figure S1A–C). Also, in H9C2 cells lines, we observed LIPUS treatment with 0.25 W/cm^2^ showed obvious improved cell viability and reactive oxygen species (ROS) generation (Figure S1A,D–F). Therefore, we selected 0.25 W/cm^2^ as the optimal parameter for subsequent experiments. Moreover, flow cytometric analysis of the treated AC16 cell line indicated that LIPUS decreased cell apoptosis in response to I/R (Figure S1G). In addition, NMCMs pretreated with I/R were also used, and no differences were observed under normal conditions with or without LIPUS therapy. However, LIPUS treatment effectively improved I/R‐mediated cellular injury (Figure S1H).

Additionally, we measured the effects of LIPUS on regulating cellular survival status under I/R conditions in endothelial cells, which represent the largest cell subset in cardiac tissue aside from cardiomyocytes. Results from human umbilical vein endothelial cells (HUVEC) experiments were consistent with those from cardiomyocyte experiments, indicating that LIPUS could improve cell viability of HUVEC (Figure S1I,J S1–S3).

Overall, these results suggested that LIPUS is an advantageous method for promoting cell survival and could retard cell apoptosis in response to I/R.

### 3.2 LIPUS ameliorates MIRI and cardiac dysfunction in mice

**FIGURE 1:**
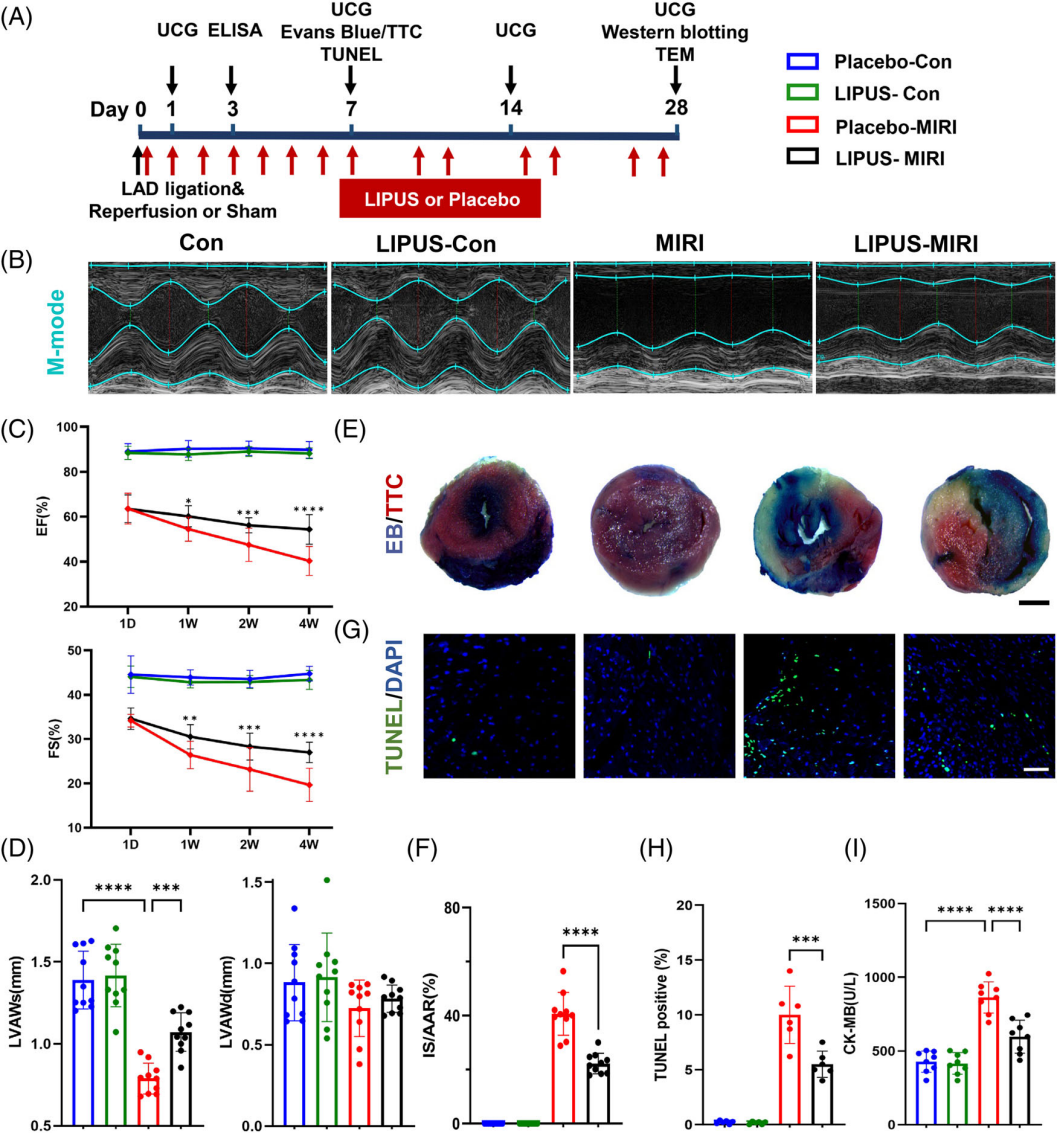
Low‐intensity pulsed ultrasound (LIPUS) therapy ameliorated myocardial ischaemia‒reperfusion injury (MIRI) and cardiac dysfunction in mice. (A) Study flowchart. (B‒D) Representative M‐mode echocardiograms. Graphs showing the time course of left ventricular ejection fraction (LVEF) (1W: *p* = 0.0406; 2W: *p* = 0.00004; 4W: *p* < 0.0001) and left ventricular fractional shortening (LVFS) (1W: *p* = 0.0052; 2W: *p* = 0.0002; 4W: *p* < 0.0001; ^*^MIRI‐no LIPUS group vs. MIRI + LIPUS group), left ventricular end‐systolic anterior wall thickness (LVAWs) (*p* = 0.0009) and left ventricular end‐diastolic anterior wall thickness (LVAWd) 4 week after MIRI (*n* = 10). (E and F) Representative images of Evans blue/TTC staining and quantification of infarct size (IS) (scale bar, 1 mm, *p* = 0.0007, *n* = 10). (G and H) TUNEL immunofluorescence staining identifies apoptotic cells, followed by 4',6‐diamidino‐2‐phenylindole (DAPI) staining (scale bar, 100 µm, *n* = 6). (I) Serum creatine kinase isoenzymes (CK‐MB) levels at 3 days after MIRI onset (*n* = 8). Results are expressed as mean ± standard deviation (SD). Comparisons of parameters were performed with analysis of variance (ANOVA) followed by Tukey's test for multiple comparisons; ^***^ *p* <0.001; ^****^ *p* < 0.0001.

To explore the therapeutic effect of LIPUS on MIRI and cardiac dysfunction in a mouse model, we attached the LIPUS probe to the chest of mice using an agar phantom gel. The LIPUS group received treatment every day from day 0 to day 7, twice a week during weeks 2−4, three times a day for 20 min each time under inhalation anesthesia with 1.2% isoflurane, while the LIPUS‐free group underwent the same procedures, including anaesthesia, but without LIPUS therapy (Figure 1A). In the control groups, the LIPUS therapy group showed no difference from the sham group. While in the MIRI group, LIPUS treatment markedly alleviated MIRI‐induced cardiac dysfunction, as manifested by increased left ventricular ejection fraction, fractional shortening and systolic anterior wall thickness (Figure 1B–D). Evans blue/triphenyltetrazolium chloride (TTC) double staining revealed that the area of myocardial infarction in mice with MIRI was significantly larger than that in the control groups, whereas irradiation with LIPUS reduced the area of myocardial infarction (Figure 1E,F). TUNEL analysis showed that MIRI significantly increased apoptosis, but LIPUS decreased the apoptosis level (Figure 1G,H). Plasma creatine kinase isoenzymes (CK‐MB) release significantly increased in the MIRI group; however, LIPUS significantly reduced this release (Figure 1I). Taken together, our results confirm that LIPUS is a beneficial therapy for ameliorating MIRI severity in mice.

### 3.3 LIPUS improves mitochondrial dysfunction and maintains mitochondrial homeostasis post‐I/R

**FIGURE 2:**
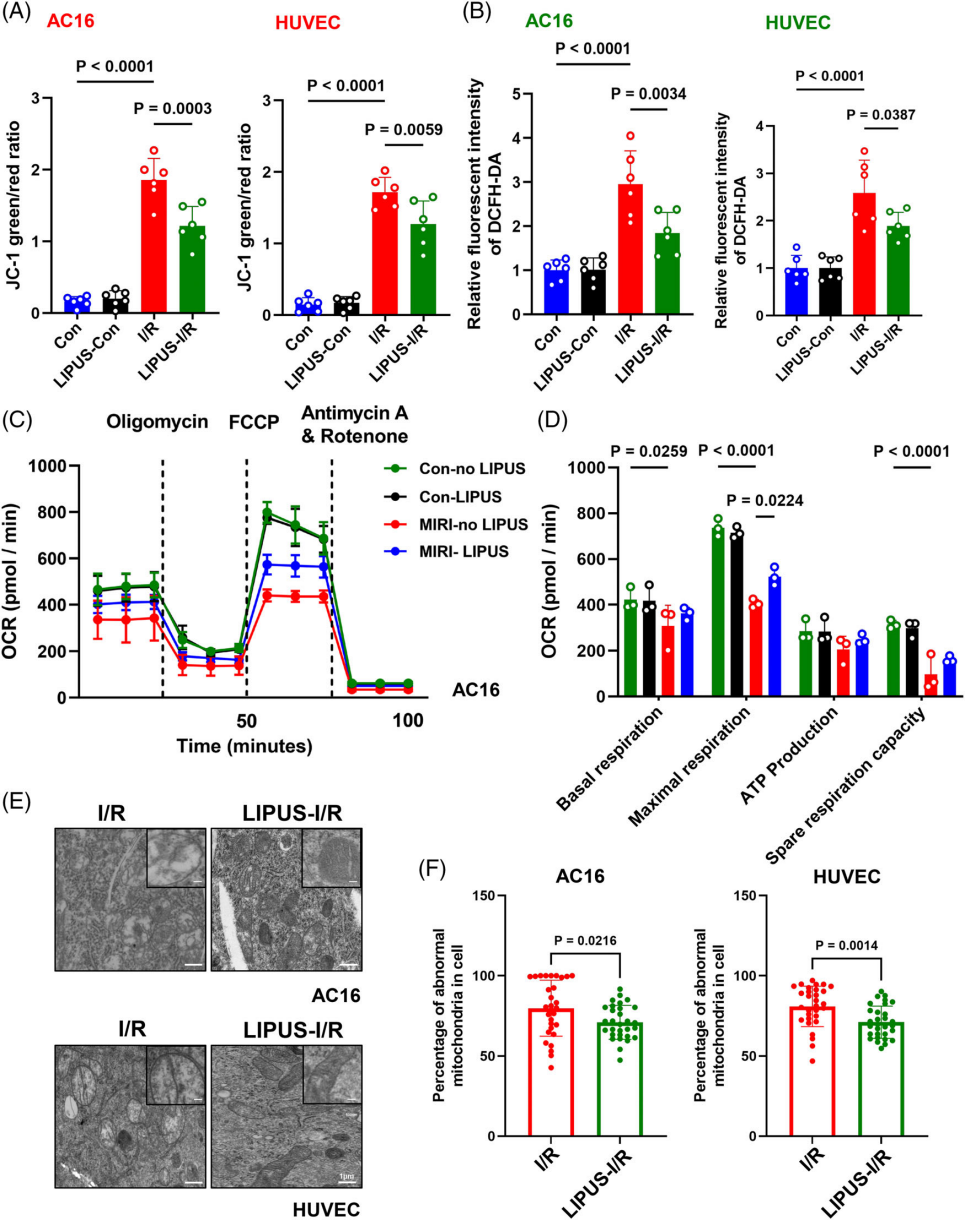
Low‐intensity pulsed ultrasound (LIPUS) therapy refined mitochondrial homeostasis in vitro. (A) The percentage of JC‐1 green/red ratio in AC16 and HUVEC cells were calculated. (B) The production of reactive oxygen species (ROS) was measured in AC16 and HUVEC cells using a fluorescence microplate reader to determine the fluorescence intensity of 2,7‐dichlorodihydrofluorescein diacetate (DCFH‐DA) (A and B, *n* = 6). (C) Cellular oxygen consumption rate (OCR) levels at the indicated times from the basal level following the presence of the indicated drugs. Oligomycin, carbonyl cyanide 4‐(trifluoromethoxy) phenylhydrazone (FCCP), antimycin A and rotenone. (D) Indices of mitochondrial respiration calculated according to the OCR profiles: basal respiration, maximal respiration, adenosine triphosphate (ATP) production and spare respiration capacity in AC16 cells (*n* = 3). (E) Representative transmission electron microscopy (TEM) images of AC16 cells and HUVECs. Scale bar, 1 µm. Upper right panels, enlarged region of interest (ROI). Scale bar, 500 nm. (F) Quantification of abnormal mitochondria in the cells. *n* = 30 cells from three independent experiments. Results are expressed as mean ± standard deviation (SD). Comparisons of parameters were performed with analysis of variance (ANOVA) followed by Tukey's test for multiple comparisons in (A‒D). Two‐tailed unpaired *t*‐tests were used for statistical analysis in (E) and (F).

Considering that mitochondrial dysfunction is a critical driver of cardiac damage caused by I/R,^7^ we examined mitochondrial status and function after LIPUS treatment. The results showed that LIPUS improved mitochondrial membrane potential (ΔΨm) and attenuated ROS generation in AC16, H9C2 and HUVEC cells under I/R conditions (Figures 2A,B and S2A–C). In addition, the oxygen consumption rate (OCR) indicated that I/R drastically suppressed mitochondrial oxygen consumption, while LIPUS treatment improved I/R‐induced mitochondrial energy metabolism injury, as measured by mitochondrial respiration analysis (Figure 2C,D). Transmission electron microscopy (TEM) analysis revealed that damaged mitochondria evidently accumulated in I/R cells, with a condensed matrix and swollen cristae observed in the majority of mitochondria. However, after LIPUS treatment, mitochondrial status was greatly improved, with a distinct reduction in the condensed matrix and swollen cristae, implying that LIPUS facilitates the excretion of damaged mitochondrial (Figure 2E,F). These findings indicated that LIPUS can ameliorate mitochondrial dysfunction and promote mitochondrial homeostasis post‐I/R.

### 3.4 Formation of migrasomes is promoted by LIPUS mechanical stimulation

LIPUS exerts an exogenous mechanical stimulation on cells, which could lead to cell vibration and displacement.^15^ Therefore, we examined the effect of LIPUS on HUVEC and AC16 cell displacement. We evaluated HUVEC migration using Transwell and wound healing assays and found that the LIPUS‐exposed groups exhibited increased cell migration into the well or wound areas (Figure S2D–G). Similarly, we transfected cells with TSPAN4‐GFP, which labels migrasomes. Confocal live cell imaging showed that LIPUS promoted cell displacement along with the increased number of migrasomes (Figure S3A,B). Notably, migrasomes are newly discovered organelles whose formation correlates with cell displacement persistence and speed capacity to mediate the removal of damaged mitochondria.^12^ Moreover, mitochondrial lateral transfer plays an important role in maintaining cardiomyocyte survival and myocardial function.^7^, ^8^ Therefore, we next investigated whether LIPUS protect MIRI via stimulating migrasomes.

**FIGURE 3:**
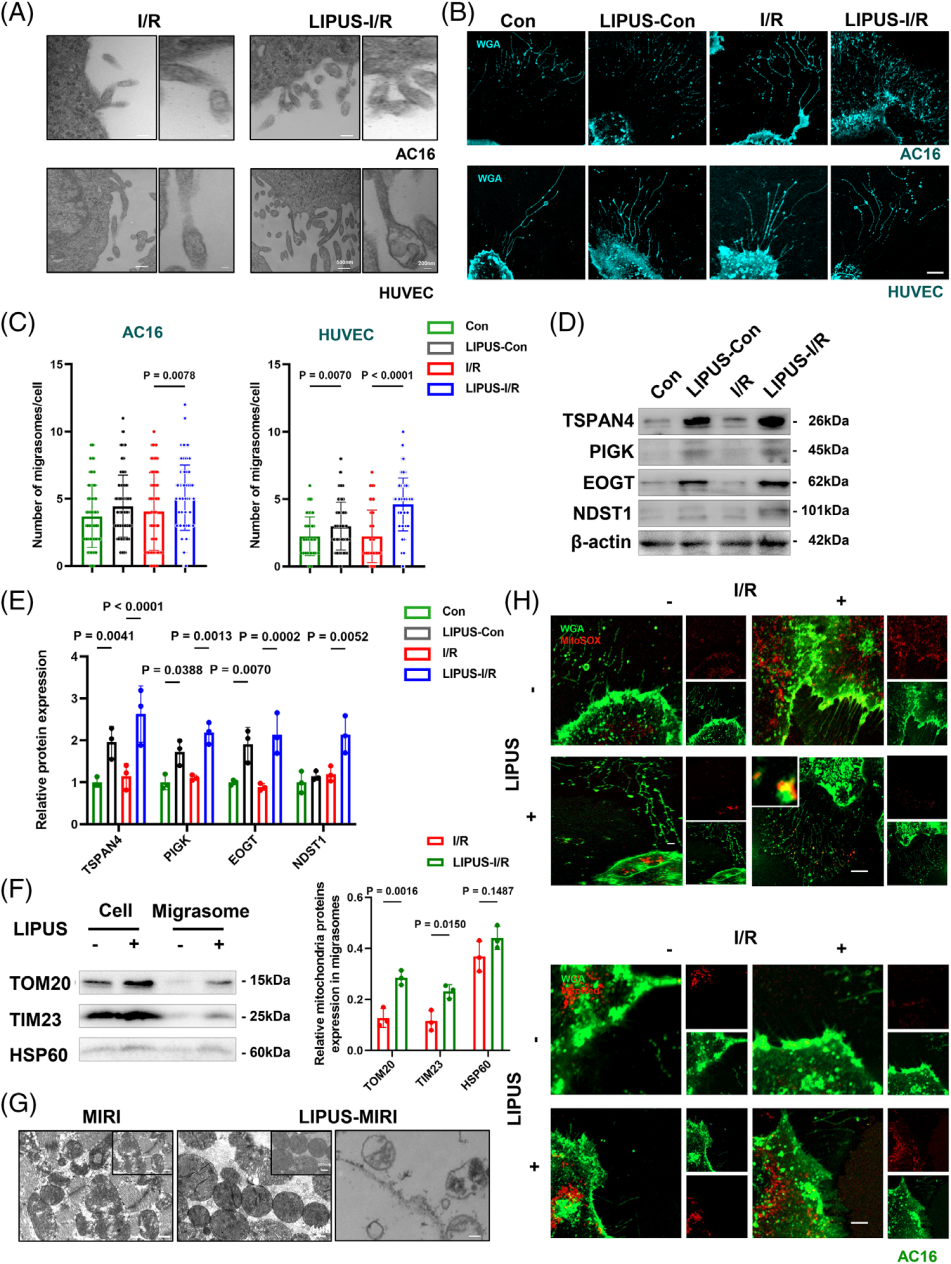
Low‐intensity pulsed ultrasound (LIPUS) facilitated migrasomes formation and damaged mitochondria expelling in a migrasome‐regulated manner in vitro. (A) Representative transmission electron microscopy (TEM) images of AC16 cells and HUVECs. Scale bar, 500 nm. Right panels, enlarged ROI. Scale bar, 200 nm. (B and C) AC16 cells and HUVECs were stained with WGA for migrasomes visualisation. Representative images of confocal microscopy and the migrasomes number of each cell. *n* = 120. Scale bar, 20 µm. (D and E) Western blot analysis for the migrasomes markers of indicated AC16 cells (*N* = 3 independent repeats). (F) Western blot analysis for the mitochondrial related proteins of indicated cardiomyocytes and migrasomes isolated from AC16 cells (*N* = 3 independent repeats). (G) Representative TEM images of cardiac tissue. Scale bar, 1 µm. Upper right panels, enlarged ROI. Scale bar, 500 nm. (H) Confocal image of AC16 cells stained with WGA, MitoSOX or MitoRed. Scale bar, 20 µm. Results are expressed as mean ± standard deviation (SD). Comparisons of parameters were performed with analysis of variance (ANOVA) followed by Tukey's test for multiple comparisons.

Our study visually demonstrated that the number of migrasomes was greatly increased in the LIPUS‐irradiated groups by TEM and confocal microscopy images (Figures 3A–C and S3C,D). Additionally, the expression levels of migrasome‐specific protein markers, including TSPAN4, PIGK, EOGT and NDST1, were significantly increased in LIPUS‐treated AC16 cells (Figure 3D,E), indicating that LIPUS indeed promoted the formation of migrasomes. Notably, in normal control group without I/R, LIPUS irradiation also modestly increased the formation of migrasomes (Figure 3D,E). Moreover, myocardium exposed to LIPUS therapy also increased the expression of migrasome‐specific protein markers under MIRI condition, but not control mice (Figure S3E). In conclusion, LIPUS enhances cell movement and promotes the formation of migrasomes.

### 3.5 LIPUS facilitates damaged mitochondria excretion via migrasomes

Next, we isolated migrasomes with or without LIPUS treatment. We found that proteins from the outer mitochondrial membrane, the inner mitochondrial membrane and the matrix accumulated strongly expressed in the migrasomes, implying that LIPUS induced an increase of mitochondria‐associated proteins in migrasomes (Figure 3F). TEM analysis of cardiac tissue revealed that after MIRI, damaged mitochondria evidently accumulated inside the migrasomes. However, after LIPUS treatment, the status of mitochondria was improved, as observed with more structured mitochondrial arrangement. Moreover, we observed migrasomes in the myocardial of LIPUS‐MIRI group, suggesting that impaired mitochondria were selectively disposed of by mitocytosis after LIPUS treatment (Figure 3G). To directly verify this observation, we stained cells with MitoRed, a marker of activated mitochondria, and MitoSOX, a marker of damaged mitochondria. We found that in comparison with the control group, I/R caused the accumulation of the MitoSOX signal and decreased the MitoRed signal in cells. However, LIPUS enhanced the MitoSOX signal inside migrasomes and inverted the distribution of the signal in cells, suggesting that LIPUS stimulates the expulsion of damaged mitochondria wrapped by migrasomes (Figures 3H and S4A). Overall, LIPUS facilitates the excretion of damaged mitochondria via migrasomes post‐I/R.

### 3.6 Migrasomes are indispensable for LIPUS protecting MIRI in mice

**FIGURE 4:**
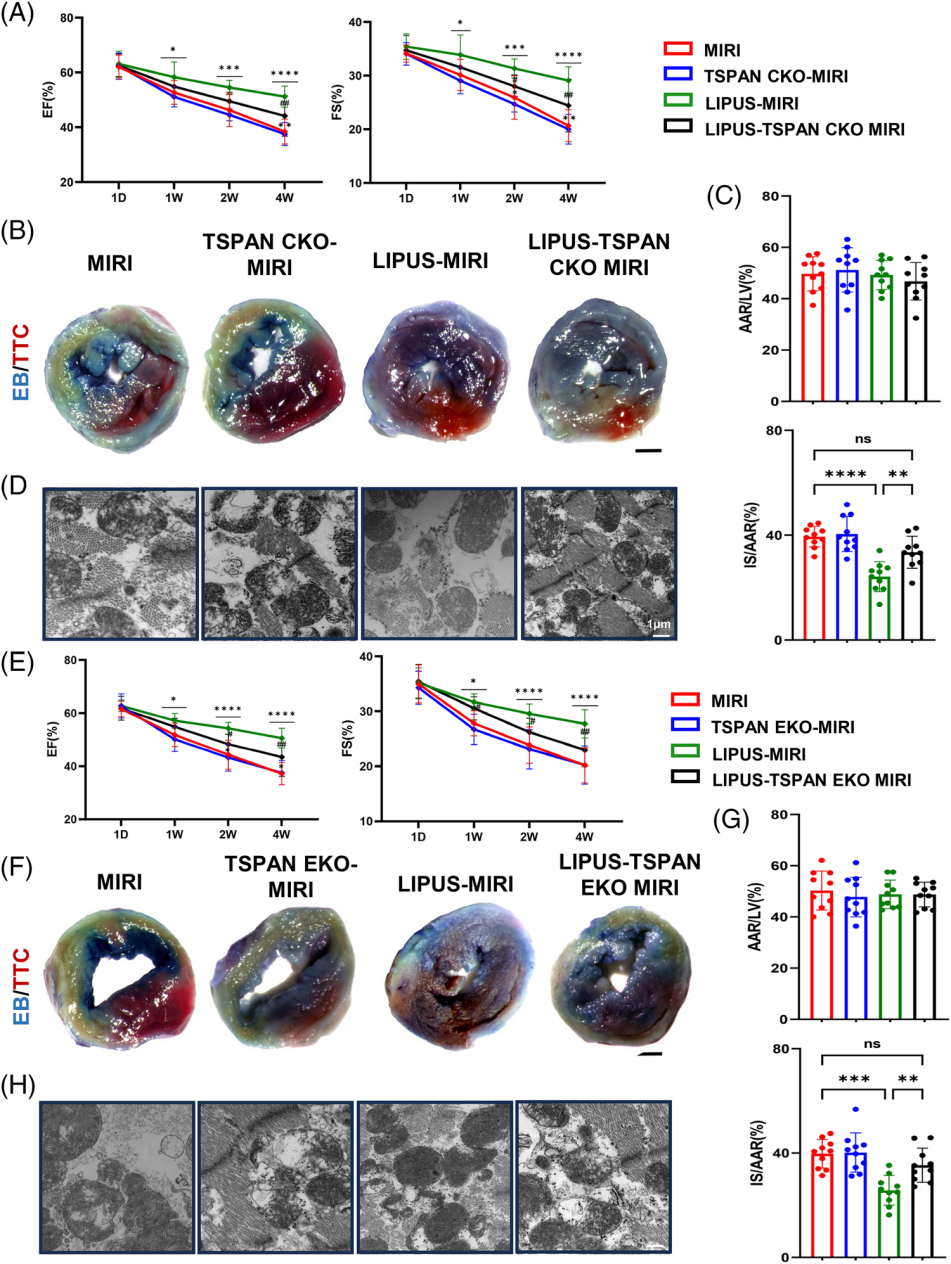
Inhibition of migrasome formation weakened the therapeutic effect of low‐intensity pulsed ultrasound (LIPUS) on myocardial ischaemia‒reperfusion injury (MIRI). (A) Echocardiogram measurement in LIPUS‐TSPAN CKO MIRI mice treated with LIPUS (left ventricular ejection fraction [LVEF]—1W: ^*^ *p* = 0.0316; 2W: ^*^ *p* = 0.0004; 4W: ^*^ *p* < 0.0001; ^*^ *p* = 0.0069; ^#^ *p* = 0.0032; left ventricular fractional shortening [LVFS]—1W: ^*^ *p* = 0.0196; 2W: ^*^ *p* = 0.0002; ^*^ *p* = 0.0452; ^#^ *p* = 0.0434; 4W: ^*^ *p* < 0.0001; ^*^ *p* = 0.0037; ^#^ *p* = 0.0017, *n* = 10). (B and C) Representative images of Evans blue/TTC staining and quantification of area at risk (AAR) and infarct size (IS) in LIPUS‐TSPAN CKO MIRI mice treated with LIPUS (scale bar, 1 mm, *p* = 0.043, *n* = 10). (D) Representative transmission electron microscopy (TEM) images of cardiac tissue. Scale bar, 1 µm. (E) Echocardiogram measurements in LIPUS‐TSPAN EKO MIRI mice treated with LIPUS (LVEF—1W: ^*^ *p* = 0.0150; ^#^ *p* = 0.0161; 2W: ^*^ *p* < 0.0001; ^#^ *p* = 0.0451; 4W: ^*^ *p* < 0.0001; ^#^ *p* = 0.0014, *n* = 10). (F and G) Representative images of Evans blue/TTC staining and quantification of AAR and IS in LIPUS‐TSPAN EKO MIRI mice treated with LIPUS (scale bar, 1 mm, *p* = 0.0001; *p* = 0.0086, *n* = 10). (H) Representative TEM images of cardiac tissue. Scale bar, 1 µm. Results are expressed as mean ± standard deviation (SD). Comparisons of parameters were performed with analysis of variance (ANOVA) followed by Tukey's test for multiple comparisons (^*^ *p* < 0.05; ^***^ *p* < 0.001; ^****^ *p* < 0.0001; ^**^ *p* < 0.01; ^***^ *p* < 0.001; ^#^ *p* < 0.05; ^##^ *p* < 0.01; ^*^MIRI group vs. LIPUS‐MIRI group; ^*^TSPAN C(E)KO‐MIRI group vs. LIPUS‐TSPAN C(E)KO MIRI group; ^#^LIPUS‐MIRI group vs. LIPUS‐TSPAN C(E)KO MIRI group).

TSPAN4 is required for migrasome formation. We examined the effects of TSPAN4 KO on migrasome formation in HUVECs by fluorescence confocal microscopy and found that the formation of migrasomes was reduced in TSPAN4 KO HUVECs (Figure S4B). To provide direct evidence to verify whether migrasomes are indeed relevant to the efficacy of LIPUS in vivo, we generated cardiomyocyte and endothelial cell‐specific TSPAN4 knockout mice, which prevented the formation of migrasomes in cardiomyocyte and endothelium, specifically. Echocardiography suggested that cardiac function worsened in the cardiac‐specific TSPAN4 knockout MIRI mice treated with LIPUS (Figure 4A). In addition, the curative effects of LIPUS were significantly abrogated in the cardiomyocyte‐specific TSPAN4 knockout MIRI mice in terms of myocardial infarct area and mitochondrial damage (Figure 4B–D). Similarly, the cardiac function was reduced in endothelial cell‐specific TSPAN4 knockout MIRI mice treated with LIPUS (Figure 4E), and the effects of LIPUS were weakened in myocardial infarct area and myocardial injury (Figure 4F–H), indicating that the impairment of migrasome formation weakens the therapeutic effect of LIPUS on MIRI.

These findings uncover that migrasomes are crucial for LIPUS therapy to protect against MIRI and ameliorate of cardiac dysfunction and mitochondrial injury.

### 3.7 LIPUS promotes RhoA/Myosin II activation and induces cytoskeletal tension to facilitate migrasomes formation

**FIGURE 5:**
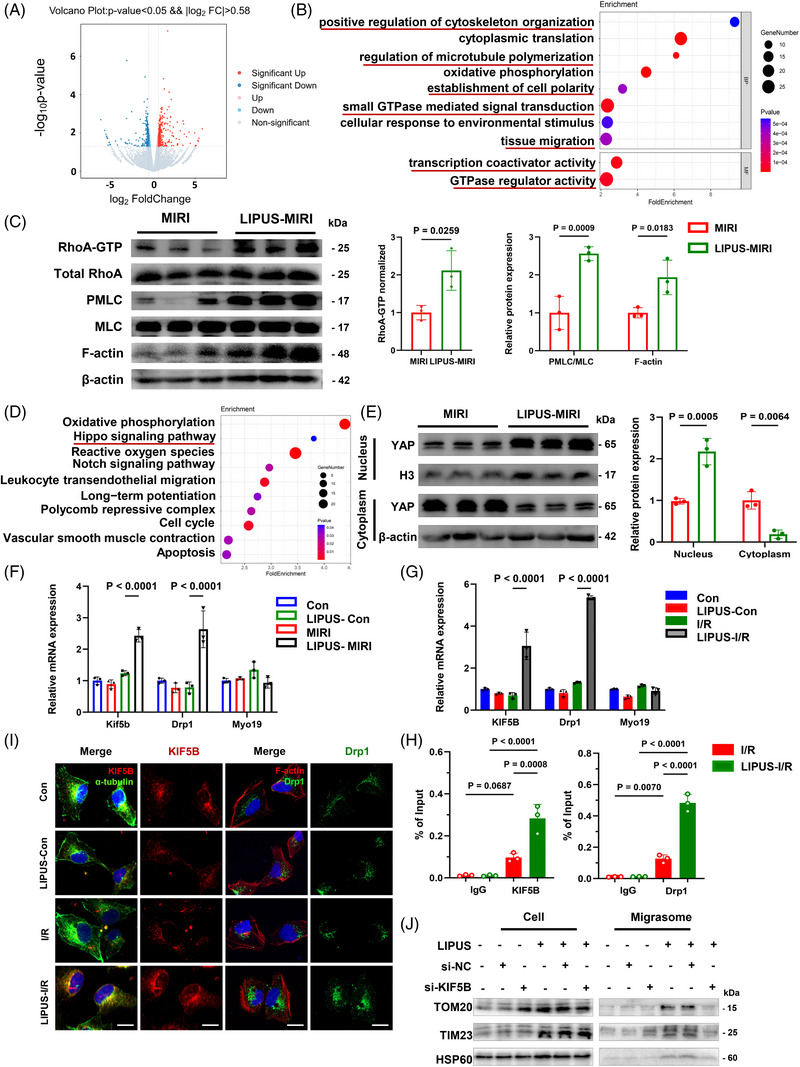
Low‐intensity pulsed ultrasound (LIPUS) evoked RhoA/Myosin II/F‐actin activation and YAP nucleation for KIF5B and Drp1 transcripting. (A) Volcano plot displaying differentially expressed genes (DEGs) between AC16 cells post‐I/R irradiated with or without LIPUS. Upregulated and downregulated DEGs are highlighted in red and green, respectively. FC, fold change. (B) Results of the gene set Gene Ontology (GO) enrichment analysis of the RNA sequencing data. (C) Western blot analysis of myocardial ischaemia‒reperfusion injury (MIRI) mice treated with or without LIPUS (*N* = 3 independent repeats). (D) Results of the gene set Kyoto Encyclopedia of Genes and Genomes (KEGG) enrichment analysis of the RNA sequencing data. (E) Western blot analysis of MIRI mice treated with or without LIPUS (*N* = 3 independent repeats). (F and G) mRNA expression analysis by RT‐qPCR in cardiac tissue of MIRI mice and AC16 cells post‐I/R and treated with LIPUS (*n* = 3). (H) Drp1 and KIF5B occupancy analysis by anti‐TEAD and ChIP‐qPCR in AC16 cells (*n* = 3). (I) Immunofluorescence co‐staining with KIF5B (red) and microtublin (green) in AC16 cells. Immunofluorescence staining with Drp1 (green) and F‐actin (red) in AC16 cells. Scale bar, 50 µm. (J) Western blot analysis for the mitochondrial related proteins of indicated cardiomyocytes and migrasomes isolated from cardiomyocytes with or without KIF5B si‐RNA transfection in AC16 cells (*N* = 3 independent repeats). Results are expressed as mean ± standard deviation (SD). Two‐tailed unpaired *t*‐tests were used for statistical analysis in (A) and (B). Comparisons of parameters were performed with analysis of variance (ANOVA) followed by Tukey's test for multiple comparisons in (C) and (D).

To discover the impact of LIPUS on regulation gene expression, we performed RNA sequencing in AC16 cells post‐I/R irradiated with and without LIPUS. In total, we identified 339 upregulated and 247 downregulated differentially regulated genes (Figure 5A). Gene Ontology analysis revealed that the differentially regulated genes were predominantly enriched in showing small GTPase mediated signal transduction, positive regulation of cytoskeletal organisation, regulation of microtubule (MT) polymerisation, establishment of cell polarity, etc. (Figure 5B).

The small GTPase RhoA is known to regulate MT polymerisation, cytoskeletal organisation and cell polarity. Moreover, RhoA activation is sensitive to mechanical stimuli and can promote Myosin II and F‐actin tension, leading to cytoskeletal rearrangement and cell movement, which could accelerate the subsequent formation of migrasomes.^18^, ^20^, ^21^, ^23^ In our study, we found that LIPUS treatment boosted the expression of RhoA‐GTP and enhanced downstream pMLC (Myosin II is activated by pMLC) in MIRI mice cardiac tissue. Moreover, we observed increased F‐actin density in LIPUS‐exposed MIRI mice (Figure 5C). Immunofluorescence analysis also revealed that LIPUS irradiation increased the fluorescence intensity of F‐actin. Additionally, treatment with Rhosin, a RhoA inhibitor, decreased the LIPUS‐induced F‐actin expression, suggesting that LIPUS promotes RhoA‐dependent cytoskeletal rearrangement through RhoA (Figure S5A). In addition, treatment with Blebbistatin, a type of Myosin II inhibitor, mitigated the F‐actin‐promoting effect of LIPUS, suggesting that LIPUS activates RhoA/Myosin II to promote F‐actin transformation (Figure S5B).

These results suggested that LIPUS induces cytoskeletal rearrangement through RhoA/Myosin II, which may explain how LIPUS promotes migrasome formation.

### 3.8 LIPUS activates YAP nuclear translocation to transcriptionally activate KIF5B and Drp1 for mitocytosis

To elucidate the downstream pathway of LIPUS, we performed Kyoto Encyclopedia of Genes and Genomes analysis and uncovered the significant enrichment of mechanically sensitive Hippo pathways (Figure 5D). YAP of the identified genes in the Hippo pathway is a transcription coactivator known for its role in converting biophysical inputs into gene expression signatures during myocardial injury.^25^ Furthermore, YAP nucleus transposition could be induced by F‐actin.^26^ Thus, we sought to investigate whether LIPUS modulates YAP activation to trans‐activation downstream gene expression. We verified the expressional changes of YAP in response to LIPUS by using Western blotting. Notably, YAP was mostly localised in the cytoplasm post‐MIRI, whereas the LIPUS‐treated group exhibited a high nuclear/cytoplasmic YAP ratio, suggesting that LIPUS activated the nuclear transport of YAP (Figure 5E). Taken together, these results suggested that LIPUS regulates YAP activity.

Yu et al. suggested that motor proteins, including KIF5B, Drp1 and Myo19, are required for mitocytosis.^12^ In our study, qRT‐PCR analysis revealed that the mRNA expression levels of KIF5B and Drp1 increased in MIRI and cells post‐I/R after treatment with LIPUS for 24 h (Figure 5F,G and Tables S1). KIF5B, a member of the kinesin family (KIFs), is a motor protein that transports organelles along MT filaments, playing an important role in transporting unhealthy mitochondria outwards and maintaining mitochondrial distribution.^27^ While Drp1 facilitates the release of KIF5B from the complex and also plays a role in mitochondrial fission to protect against cardiac ischaemia/reperfusion.^28^

Thus, we investigated whether the nuclear YAP, induced by LIPUS, affects the genes associated with mitocytosis. It has been recognised that TEAD served as a transcription factor that interacts with YAP. Moreover, we discovered TEAD binds to the promoters of the KIF5B and Drp1 genes in the JASPAR database. The chromatin immunoprecipitation assay (ChIP) assay revealed that TEAD could directly bind to the target sequences of the KIF5B and Drp1 promoter regions, suggesting that nuclear YAP assists TEAD in KIF5B and Drp1 transcription (Figure 5H and Tables S2–S3). Furthermore, we observed enhanced the intensity of Drp1 levels in AC16 cells and HUVECs after LIPUS treatment. In addition, LIPUS exacerbated cytosolic KIF5B co‐localisation with MTs, suggesting that mitochondria may be trafficked together by Drp1 and KIF5B along MTs from the cytosol towards the cell membrane and then sent into migrasomes after LIPUS stimulation (Figures 5I and S5C,D).

Next, we evaluated the role of LIPUS‐induced KIF5B expression in the regulation of mitochondrial translocation into migrasomes. Successful si‐KIF5B transfection was confirmed by Western blotting (Figure S6A). JC‐1 staining and 2,7‐dichlorodihydrofluorescein diacetate (DCFH‐DA) revealed ΔΨm loss and ROS generation in the si‐KIF5B group, implying that KIF5B silencing resulted in the recurrence of mitochondrial disorders despite LIPUS treatment (Figure S6B,C). Proteins from mitochondria that exist in migrasomes were also reduced by si‐KIF5B (Figure 5J), suggesting that KIF5B plays an important role in the migratory function of impaired mitochondria during LIPUS therapy. Moreover, we found that si‐KIF5B transfection visibly eliminated the phenomenon that LIPUS excites damaged mitochondrial ejection with migrasomes (Figure S6D,E), indicating the crucial role of KIF5B in LIPUS‐mediated mitocytosis. Therefore, LIPUS‐induced KIF5B is a pivotal dynein that transports impaired mitochondria out of the cells into migrasomes.

Taken together, we confirmed that LIPUS activated YAP nucleus transposition post‐I/R, with important implications for mechano‐transduction and functional regulatory in upregulating KIF5B and Drp1 for migrasome‐mediated mitocytosis.

### 3.9 RhoA/Myosin II pathway is critical for LIPUS protecting MIRI and improvement of cardiac dysfunction

**FIGURE 6:**
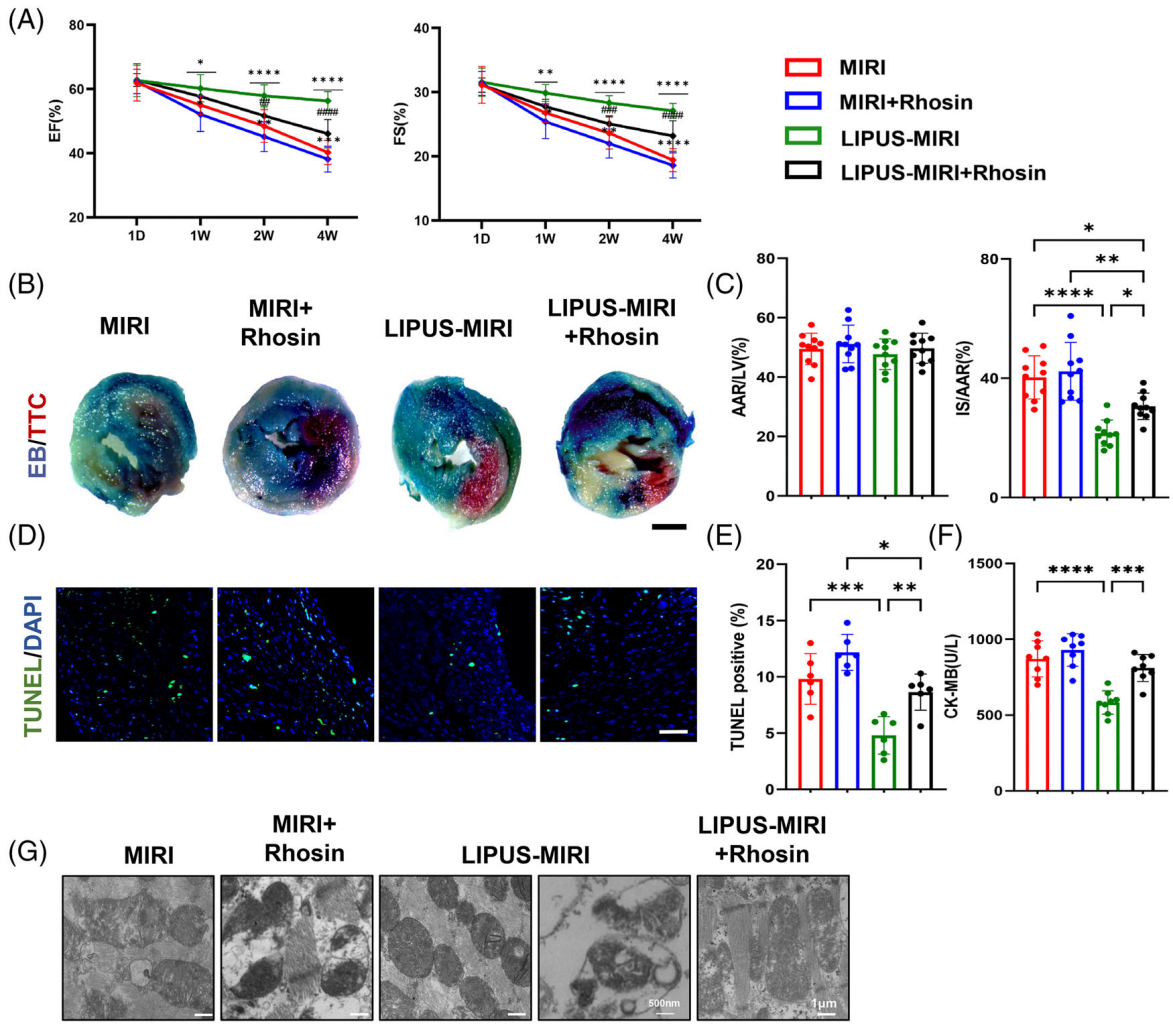
Absence of the beneficial effects of low‐intensity pulsed ultrasound (LIPUS) on myocardial ischaemia‒reperfusion injury (MIRI) improvement and damaged mitochondria ejection in mice with RhoA inhibition. (A) Echocardiogram measurements in MIRI mice treated with Rhosin and LIPUS (left ventricular ejection fraction [LVEF]—1W:^*^ *p* = 0.0235; *p = 0.0155; 2W: ^*^ *p* < 0.0001; ^*^ *p* = 0.0026; ^#^ *p* = 0.0049; 4W: ^*^ *p* < 0.0001; ^*^ *p* = 0.0001; ^#^ *p* < 0.0001; left ventricular fractional shortening [LVFS]—1W: ^*^ *p* = 0.0022; ^*^ *p* = 0.0324; 2W: ^*^ *p* < 0.0001; ^*^ *p* = 0.0024; ^#^ *p* = 0.0010; 4W: ^*^ *p* < 0.0001; ^*^ *p* < 0.0001; ^#^ *p* < 0.0001; left ventricular end‐systolic anterior wall thickness [LVAWs]—*p* = 0.0006; ^*^MIRI group vs. LIPUS‐MIRI group; *p* = 0.0226; ^*^LIPUS‐MIRI group vs. LIPUS‐MIRI + Rhosin group; *p* = 0.0252; ^*^MIRI + Rhosin group vs. LIPUS‐MIRI + Rhosin group, *n* = 10). (B and C) Representative images of Evans blue/TTC staining and quantification of area at risk (AAR) and infarct size (IS) in MIRI mice treated with Rhosin and LIPUS (scale bar, 1 mm, *p* < 0.0001; ^*^ MIRI group vs. LIPUS‐MIRI group; *p* = 0.263; ^*^LIPUS‐MIRI group vs. LIPUS‐MIRI + Rhosin group; *p* = 0.0024; ^*^MIRI + Rhosin group vs. LIPUS‐MIRI + Rhosin group; *p* = 0.015; ^*^MIRI group vs. LIPUS‐MIRI + Rhosin group, *n* = 10). (D and E) TUNEL immunofluorescence staining identifies apoptotic cells, followed by DAPI staining (scale bar, 100 µm, *p* = 0.0006; ^*^MIRI group vs. LIPUS‐MIRI group; *p* = 0.0073; ^*^LIPUS‐MIRI group vs. LIPUS‐MIRI + Rhosin group; *p* = 0.0139; ^*^MIRI + Rhosin group vs. LIPUS‐MIRI + Rhosin group, *n* = 6). (F) Serum CK‐MB levels at 3 days after MIRI onset treated with Rhosin and LIPUS. (G) Representative transmission electron microscopy (TEM) images of cardiac tissue. Scale bar, 1 µm. Enlarged ROI. Scale bar, 500 µm (*p* < 0.0001; ^*^MIRI group vs. LIPUS‐MIRI group; *p* = 0.0005; ^*^LIPUS‐MIRI group vs. LIPUS‐MIRI + Rhosin group, *n* = 8). Results are expressed as mean ± standard deviation (SD). Comparisons of parameters were performed with analysis of variance (ANOVA) followed by Tukey's test for multiple comparisons (^*^ *p* < 0.05; ^**^ *p* < 0.01; ^***^ *p* < 0.001; ^****^ *p* < 0.0001; ^**^ *p* < 0.01; ^***^ *p* < 0.001; ^****^ *p* < 0.0001; ^#^ *p* < 0.05; ^####^ *p* < 0.0001; ^*^MIRI‐no LIPUS group vs. MIRI‐LIPUS group; ^*^MIRI + Rhosin‐no LIPU group vs. MIRI + Rhosin‐LIPUS group; ^#^MIRI‐LIPUS group vs. MIRI + Rhosin‐LIPUS group).

To explore the important role of LIPUS‐activated RhoA/Myosin II in improving the severity of MIRI, we used their inhibitors, Rhosin or Blebbistatin, respectively, in a MIRI mouse model while measuring the efficacy of LIPUS on MIRI. Successful RhoA inhibition by Rhosin was confirmed by Western blotting (Figure S7A). Echocardiography showed that cardiac dysfunction was attenuated by LIPUS, but was aggravated after appending Rhosin (Figure 6A). A significant decrease in the infarct size was observed in MIRI mice after LIPUS treatment. Notably, Rhosin diminished this effect (Figure 6B,C). Similarly, while LIPUS noticeably reduced MIRI‐induced apoptosis, and the CK‐MB boom, these curative changes were abrogated after Rhosin injection (Figure 6D–F), indicating that RhoA activation is a crucial component for improving MIRI by LIPUS therapy.

We also injected Blebbistatin, which intercepted the formation of migrasomes by inhibiting Myosin II, into MIRI mice. Despite LIPUS therapy, the cardiac function of Blebbistatin‐treated MIRI mice was aggravated (Figure S7B). In addition, the curative effects of LIPUS were reduced in terms of myocardial infarct area and myocardial injury (Figure S7C–G).

These results suggested that the activation of RhoA/Myosin II is involved for the effective treatment of MIRI and cardiac dysfunction with LIPUS.

### 3.10 RhoA/Myosin II are essential components for migrasome‐mediated mitocytosis induced by LIPUS

**FIGURE 7:**
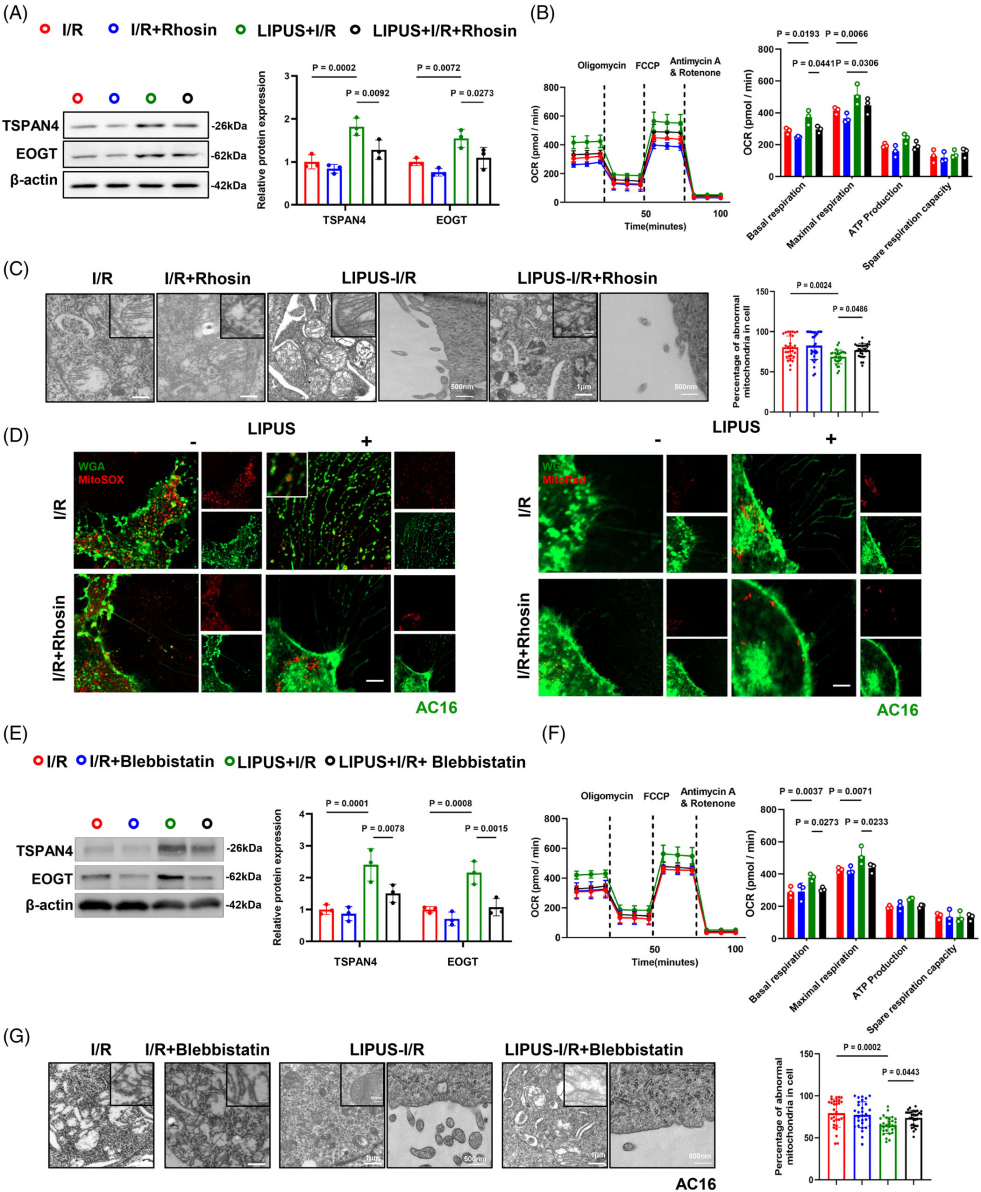
Inhibition of RhoA or Myosin II eliminated the effect of low‐intensity pulsed ultrasound (LIPUS) on mitochondrial status improvement and migrasome‐mediated mitocytosis. (A) Western blot analysis of AC16 cells treated with Rhosin and LIPUS (*N* = 3 independent repeats). (B) Oxygen consumption rate (OCR) and mitochondrial respiration of indicated AC16 cells treated with Rhosin and LIPUS (*n* = 3). (C) Representative transmission electron microscopy (TEM) images of AC16 cells and quantification of abnormal mitochondria in the cells treated with Rhosin and LIPUS. *n* = 30 cells from three independent experiments. Scale bar, 1 µm. Enlarged ROI. Scale bar, 500 nm. Enlarged ROI. Scale bar, 200 nm. (D) Confocal image of AC16 cells stained with WGA, MitoSOX or MitoRed. Scale bar, 20 µm. (E) Western blot analysis of AC16 cells treated with Blebbistatin and LIPUS (*N* = 3 independent repeats). (F) OCR and mitochondrial respiration of indicated AC16 cells treated with Blebbistatin and LIPUS (*n* = 3). (G) Representative TEM images of AC16 cells treated with Blebbistatin and LIPUS. *n* = 30 cells from three independent experiments. Scale bar, 1 µm. Enlarged ROI. Scale bar, 500 nm. Scale bar, 200 nm. Results are expressed as mean ± standard deviation (SD). Comparisons of parameters were performed with analysis of variance (ANOVA) followed by Tukey's test for multiple comparisons.

The cardiac tissue of MIRI mice vividly showed that LIPUS‐driven mitocytosis was drastically weakened by using Rhosin or Blebbistatin, as observed by TEM (Figures 6G and S7H). To further explore the important role of RhoA and Myosin II in LIPUS‐driven mitocytosis, we utilised Rhosin or Blebbistatin in vitro. We observed RhoA activity was successfully inhibited after Rhosin treatment (Figure S8A). Calcein‐AM/EthD‐1 staining and CCK8 analysis indicated a significant decrease in cell viability following Rhosin treatment (Figure 8B,C). JC‐1 and DCFH‐DA staining indicated significant mitochondrial membrane depolarisation and ROS generation following RhoA inhibition, implying that despite LIPUS treatment, RhoA inhibition results in the recurrence of mitochondrial disorders (Figure S9A,B). Furthermore, we found that the inhibition of RhoA resulted in decreased cell migration and reduced number of migrasomes in I/R cells under LIPUS stimulation (Figures 7A, S9C,D and S10A,B). The OCR observed in the Rhosin‐treated I/R cells indicated a significant repression of cell mitochondrial oxygen consumption compared to I/R cells after LIPUS irradiation (Figure 7B). Confocal microscopy and TEM images showed that mitocytosis induced by LIPUS was impeded with the accumulation of damaged mitochondria after RhoA inhibition (Figures 7C,D and S10C,D).

Furthermore, it was proved that the formation of migrasomes was inhibited by Blebbistatin treatment under LIPUS stimulation (Figure 7E). In addition, Blebbistatin effectively suppressed cell mitochondrial oxygen consumption compared to that in LIPUS‐treated I/R cells (Figure 7F). Treatment with Blebbistatin resulted in an apparent decrease in cell viability and a higher apoptosis level (Figure S11A–C). JC‐1 and DCFH‐DA staining indicated that mitochondrial dysfunction was aggravated compared with LIPUS only group (Figure S12A,B). The inhibition of Myosin II leads to reduced cell migration and a decreased number of migrasome formation in I/R cells under LIPUS stimulation (Figures S12C,D and S13A,B). Furthermore, mitocytosis was hampered in the Blebbistatin‐ and LIPUS‐treated cells compared with LIPUS‐only cells (Figures 7G and S13C,D).

Accordingly, we concluded that LIPUS‐induced RhoA/Myosin II are essential components to drive migrasome‐mediated mitocytosis.

## 4 DISCUSSION

In this study, we uncovered that LIPUS serves as an innovative and effective therapeutic strategy to ameliorate MIRI. Second, we clarified that migrasome‐mediated mitocytosis is a critical mechanism by which LIPUS promotes mitochondrial quality control, rescues mitochondrial dysfunction and alleviates MIRI. Third, we illustrated that cellular mitocytosis is dependent on LIPUS‐driven RhoA/Myosin II/F‐actin activation and YAP/Drp1/KIF5B axes.

AMI is a leading cause of human death worldwide.^1^, ^2^ Both ischaemia and reperfusion after therapeutic intervention inevitably cause cascading cycles of MIRI.^29^ Patients subjected to this succumb to myocardial dysfunction, resulting in myocardial cell death, which contributes to morbidity and mortality. Novel cardioprotective strategies aiming at reducing myocardial injury and improving patient outcomes should therefore be investigated.^30^ Several clinical medicines have been developed for the prevention and treatment of MIRI, including cyclosporine A, adenosine, exenatide and aspirin. Although they may improve prognosis, the majority of the drugs still exhibit systemic side effects.^5^, ^31^ Ischaemic preconditioning showed great value in protecting against MIRI in animal studies; however, its cardioprotective effects in clinical practice are still open to question.^32^ For now, it is imperative to establish safe, non‐invasive therapeutic means for MIRI that can achieve clinical transformation. LIPUS is one such novel, inexpensive and safe biophysical therapy. Several published studies have shown that LIPUS has the potential to protect against many cardiovascular diseases, including cardiac dysfunction in AMI. However, its role in MIRI remains unknown. In this study, we found that LIPUS improved cardiac left ventricular function, reduced infarct area and decreased cellular apoptosis in MIRI mice. These basic findings are consistent with previous research, showing the potential ability of LIPUS to protect patients suffering from MIRI.^13^, ^14^ Therefore, we emphasised that the physical stimulus of LIPUS is a potential alternative treatment to alleviate MIRI.

During the stress conditions of MIRI, the mitochondria are damaged and contribute to the development of cardiac dysfunction. The generation of ROS and the release of cytochrome C can activate a self‐amplifying caspase cascade, further leading to necrotic and apoptotic cell death.^33^ Thus, the accumulation of even small numbers of damaged mitochondria can have a drastic effect in the long term, making it clear that the removal of damaged mitochondria is critical for cell survival after I/R.^33^ Approaches to improve MIRI have shown promise in preclinical studies of mitochondrial quality control.^34^ The use of the mitochondrial permeability transition pore blocker cyclosporine A for the treatment of MIRI showed positive therapeutic effects in small‐scale trials,^35^, ^36^ while a larger multicenter studies yielded neutral results.^37^ In addition, the MITOCARE study demonstrated that TRO40303, a mitochondrial‐targeted drug did not show a positive effect in limiting reperfusion injury.^38^ Therefore, theories and approaches based on mitochondrial regulation to protect MIRI need to be further explored.

Recently, studies have unveiled that mitochondria can be transferred via vesicles to rescue endangered cardiomyocytes.^37^, ^39^ Migrasomes are newly discovered vesicles that are ultimately expelled from migrating cells. They can clear mitochondria from cells to mitigate mitochondrial stress burden. In this context, mitocytosis represents a beneficial server for mitochondrial quality control, as it combines mitochondrial homeostasis with cell migration. By continuously removing damaged mitochondria, migrasome‐mediated mitocytosis can avoid the adverse effects associated with the accumulation of dysfunctional mitochondria.^12^, ^37^ In supporting of this, our data showed that LIPUS treatment generally increases the number of migrasomes, regardless of whether cells are IR injured or not. Inhibition of migrasome formation weakens the therapeutic effect of LIPUS on MIRI by using the cardiac and endothelial‐specific TSPAN4 knockout MIRI mice. Moreover, LIPUS improves mitochondrial conditions in MIRI by promoting the formation of migrasomes that discharge damaged mitochondria. When migrasome formation is impaired, cell viability and mitochondrial potential are reduced, and damaged mitochondria accumulate, suggesting that LIPUS therapy is beneficial for MIRI via migrasome‐mediated mitocytosis. In this study, LIPUS irradiation also modestly increased the formation of migrasomes in the normal control group without I/R injury. Whereas, the effects were more pronounced in I/R group. We inferred that under I/R conditions, cells are in a state of stress and are therefore more inclined to be sensitive to LIPUS mechanical force stimulation. Therefore, I/R‐induced cells are susceptible to be modulated by LIPUS to produce migrasomes.

Additionally, we investigated the mechanism of the beneficial effects of LIPUS therapy on mitocytosis. The small GTPase RhoA plays a crucial role in coordinating various cellular processes, including the regulation of cytoskeletal rearrangements and cell polarity.^40^, ^41^, ^42^ RhoA activity and cytoskeletal tension are important players in various mechano‐transduction pathways, leading to changes in gene expression that affect cell status.^40^ Previous studies have demonstrated that RhoA activation is sensitive to mechanical stimuli.^17^, ^18^, ^19^, ^20^ Whereas RhoA tends to be active in response to MIRI, which is an endogenous cardiac protection mechanism.^24^, ^33^ However, endogenous RhoA activation is not sufficient to prevent myocardial deterioration. Investigators have attempted to over‐activate RhoA by transgene and AAV delivery. They discovered that exogenous RhoA activation attenuated MIRI in mice.^24^, ^33^ Our study demonstrated an increase in active RhoA associated with LIPUS treatment. RhoA promotes Myosin II and F‐actin tension to form the cytoskeleton and mediates actin polymerisation and density, playing a conducive role in cytoskeletal rearrangement and cell movement.^21^ This process facilitates persistent cell migration by stimulating the rapid retraction of the migrating cell posterior, the consequent formation of retraction fibers and migrasomes.^22^, ^23^ Consistently, our study showed that active Myosin II can induce F‐actin after LIPUS treatment, whereas the RhoA suppressant could reduce F‐actin formation. Furthermore, when we inhibited RhoA and Myosin II, the therapeutic effects of LIPUS, migrasome formation, and mitocytosis were decreased, indicating that RhoA‒Myosin II‐induced actin polymerisation is indispensable for the therapeutic effect of LIPUS via migrasome‐mediated mitocytosis after MIRI.

Previous studies have shown that RhoA signalling provides cardioprotection against ischaemic injury by inhibiting mitochondrial death pathways.^24^ However, mitochondrial integrity is preserved not only by inhibiting of mitochondrial death pathway, but also by mitochondrial quality control mechanisms.^43^ Our study demonstrated that LIPUS activates RhoA signalling and protects against MIRI through migrasome‐mediated mitocytosis, which pertains to a mechanism of mitochondrial quality control. Notably, the downstream pathways of RhoA are complex and may have ambiguous and dubious functions in cardiac injury.^44^ Therefore, it remains important to explore other beneficial pathways of RhoA that are associated with cardioprotective effects on MIRI in future studies. By inhibiting detrimental pathways and enhancing beneficial ones, we can identify new therapeutic targets for the treatment of MIRI.

In addition, we noted that F‐actin, which is regulated by active RhoA, participates in chromatin mechanical signalling and chromatin accessibility, and that YAP is also involved in gene transcription, which is regulated by F‐actin.^45^, ^46^ The actin cytoskeleton interacts with Myosin II to generate tension, which promotes YAP nuclear transport. Surprisingly, inhibition of actin compaction prevents the nuclear transport of YAP and cell migration,^26^, ^47^ while cytosolic retention of YAP can lead to impaired transcription of antioxidant defense genes associated with mitochondrial ROS, organelle swelling and apoptosis in an I/R model.^48^ Restoration of YAP nuclear localisation rescues mtROS, mitochondrial damage and apoptosis. Our study showed that LIPUS increased RhoA/Myosin II and F‐actin expression and promoted YAP nucleus translocation while reducing its cytoplasmic content. While inhibition of RhoA and Myosin abolished the protective role of LIPUS on MIRI. These results demonstrate the therapeutic role of LIPUS in MIRI mice is dependent on promoting active RhoA‒Myosin II‒F‐actin and YAP nuclear translocation, thereby enhancing the transcription of certain genes related to mitochondrial transport.

KIF5B is important for maintaining mitochondrial DNA integrity and function. Disruption of KIF5B results in improper distribution and abnormal perinuclear clustering of mitochondria.^27^ MTs form a dynamic network capable of shuttling cargo to proper destinations, including regulating mitochondrial movement and distribution.^8^ We show that KIF5B is accompanied by MTs to transport mitochondria under LIPUS stimulation. Silencing KIF5B abolished LIPUS‐induced mitocytosis, indicating that the mitochondrial motor protein KIF5B is a key component for LIPUS to promote the clearance of damaged mitochondria and enhance mitochondrial quality control.

Furthermore, the enhancement of KIF5B‐MT interaction depends on an appropriately high level of Drp1, which triggers the displacement of KIF5B from the kinesin‐1 complex, increasing its binding to MT tracks and mitochondrial transport.^28^ Drp1 alone can translocate to the mitochondria and generate the force necessary for mitochondrial fission, which occurs prior to apoptosis and has been suggested to protect against cell apoptosis and death.^49^ Cardiac‐specific Drp1 knockout or mutation results in mitochondrial dysfunction, cardiomyocyte death and increased infarct from I/R, indicating that Drp1 is an adaptive molecular under stress stimulation.^50^ Here, we confirmed that YAP nuclear translocation assists TEAD to enhance Drp1 and KIF5B gene transcription. Besides, Drp1 promotes KIF5B, which transports damaged mitochondria along the MTs into the extracellular migrasomes. This explains how LIPUS promotes the excretion of damaged mitochondria from cells into migrasomes.

There are several limitations that should be acknowledged here. First, this study primarily focused on a mouse model, whereas LIPUS has been shown to improve cardiac dysfunction in a porcine model of chronic myocardial ischaemia. Therefore, the findings of our study need to be validated in large animal models to further interpret their therapeutic effects and promote their clinical application. Second, we administered LIPUS therapy every day for the entire first week, and then twice a week from the second to the fourth week. However, it is still not clear whether this is the optimal schedule and whether a longer therapeutic period or optimised frequency would achieve better efficacy. Further validation in large animal models and optimisation of therapeutic regimens may be required to fully realise the potential of LIPUS therapy for the treatment of MIRI. Moreover, the in vitro supporting data are from AC16 and NMCM, which are not highly relevant to adult cardiomyocytes in vivo. Although the present study focused on mitocytosis as the main mechanism of the LIPUS‐induced beneficial effects in the present study, other mechanisms of LIPUS therapy on MIRI remained to be investigated. These include changes in mitochondrial dynamics and other forms of mitochondrial quality control. The activation of Yap by LIPUS also appears to have an effect on cardiac regeneration or fibrosis that needs to be further explored. Additionally, the damaged mitochondria are not only transported outward by KIF5B but also avoid binding to the inward motor proteins. However, the role of LIPUS in regulating inward motor proteins during mitocytosis remains unclear. Finally, mitocytosis may be more pronounced in cells with stronger movement ability; therefore, follow‐up studies should be extended to other types of migratory cells, such as T lymphocytes and monocytes.

## 5 CONCLUSION

In conclusion, we have demonstrated that LIPUS therapy is a promising method for the treatment of MIRI. Functionally, LIPUS therapy attenuated cardiac infarct area, left ventricular dysfunction and apoptosis. Mechanistically, LIPUS activated RhoA/Myosin II and polymerised microfilament F‐actin to promote the migrasome production and cell movement. Furthermore, LIPUS induced YAP nuclear transport and Drp1 and KIF5B transcriptional activation, which directly promoted the excretion of damaged mitochondria and enhanced mitochondrial quality control in MIRI. Overall, this study provides a basis for the development of a new non‐invasive therapy for patients with MIRI, which has the potential to transform and guide clinical treatment.

## AUTHOR CONTRIBUTIONS

Ping Sun, Yifei Li, Weidong Yu, Jianfeng Chen, Zhuo Wang, Pingping Wan, Shuai Fu, Ge Mang, Stephen Choi, Zhuo Du, Caiying Tang, Song Li and Guoxia Shi performed research. Ping Sun, Yifei Li, Maomao Zhang and Chao Wang analysed data. Ping Sun, Yifei Li, Jiawei Tian, Jiannan Dai and Xiaoping Leng designed the study and wrote the manuscript. All authors have read and approved the article.

## CONFLICT OF INTEREST STATEMENT

The authors declare they have no conflicts of interest.

## DATA AVAILABITY STATEMENT

All data were available from the corresponding author upon reasonable request.

## ETHICS STATEMENT

All animal experiments and protocols were approved by the Committee on the Ethics of Animal Experiments of the Second Affiliated Hospital of Harbin Medical University (approval no. sydwgzr2021‐139).

## Supporting information

Supporting Information
