# Supplementary material for: Low‐intensity pulsed ultrasound improves myocardial ischaemia‒reperfusion injury via migrasome‐mediated mitocytosis
Source: Clin Transl Med. 2024 Jul 1;14(7):e1749. doi: 10.1002/ctm2.1749 (PMC11216834; doi:10.1002/ctm2.1749)
Supplement: Supplementary file 1 — Supporting Information [file CTM2-14-e1749-s001.docx]

**SUPPLEMENTAL MATERIAL**

**Low-Intensity Pulsed Ultrasound Improves Myocardial Ischemia-Reperfusion Injury via Migrasome-Mediated Mitocytosis**

Ping Sun^1,2,3#^, Yifei Li^1,2,3#^, Weidong Yu^1,2#^, Jianfeng Chen^2,4#^, Pingping Wan^3,5^, Zhuo Wang^1,2,3^, Maomao Zhang^3,5^, Chao Wang^1,2,3^, Shuai Fu^1,2,3^, Ge Mang^3,5^, Stephen Choi^6^, Zhuo Du^3,5^, Caiying Tang^3,5^, Song Li^3,5^, Guoxia Shi^3,5^, Jiawei Tian^1,2*^, Jiannan Dai^3,5*^, Xiaoping Leng^1,2*^

^1^ Department of Ultrasound, the Second Affiliated Hospital of Harbin Medical University, Harbin, Heilongjiang Province, China; ^2^ Ultrasound Molecular Imaging Joint Laboratory of Heilongjiang Province, Harbin, Heilongjiang Province, China; ^3^ The Key Laboratory of Myocardial Ischemia, Harbin Medical University, Ministry of Education, Harbin, Heilongjiang Province, China; ^4^ Laboratory Animal Center, the Second Affiliated Hospital of Harbin Medical University, Harbin, China; ^5^ Department of Cardiology, the Second Affiliated Hospital of Harbin Medical University, Harbin, Heilongjiang Province, China; ^6^SXULTRASONIC Ltd. Kerry Rehabilitation Medicine Research Institute, Shenzhen, Guangdong Province, China.

^#^ These authors contributed equally to this work.

***Corresponding author:**

Jiawei Tian

Department of Ultrasound, the Second Affiliated Hospital of Harbin Medical University.

Ultrasound Molecular Imaging Joint Laboratory of Heilongjiang Province.

Harbin 150086, China; Tel: +86 0451 86605811; Email: [jwtian@hrbmu.edu.cn](mailto:jwtian@hrbmu.edu.cn);

Jiannan Dai

The Key Laboratory of Myocardial Ischemia, Harbin Medical University, Ministry of Education.

Department of Cardiology, the Second Affiliated Hospital of Harbin Medical University.

Harbin 150086, China; Tel: +86-451-86605359; Email: [daijiannandr@163.com](mailto:daijiannandr@163.com);

Xiaoping Leng

Department of Ultrasound, the Second Affiliated Hospital of Harbin Medical University.

Ultrasound Molecular Imaging Joint Laboratory of Heilongjiang Province.

Harbin 150086, China; Tel: +86 0451 86605811; Email: [xpleng@ems.hrbmu.edu.cn](mailto:xpleng@ems.hrbmu.edu.cn).

**Rhosin and Blebbistatin Administration**

For *in vivo* experiment, the inhibitors were prepared in 5% DMSO and 0.9% normal saline solution, and the powder was dissolved by gentle heating. Successively add DMSO (10%), Tween (5%) and saline (45%) to PEG300 (40%) by volume. The prepared Rhosin (HY-12646/CS-3877, MCE) and Blebbistatin (HY-13813/CS-0007920, MCE) solution were injected intraperitoneally (i.p.) at 40 mg/kg and 45 mg/kg 15 minutes before LIPUS treatment each time for 7 consecutive days, respectively.

For *in vitro* experiment, cells were planted and incubated in six-well plates with medium containing 30 μM Rhosin or 10 μM Blebbistatin for 2 days.

**Echocardiography**

After anesthetizing mice with 1-2% isoflurane at 1 day and 1, 2, and 4 weeks after surgery respectively, transthoracic echocardiography was performed using a VisualSonics Vevo 3100 ultrasound (VisualSonics Inc., Toronto, ON, Canada). Left ventricular end-systolic anterior wall thickness (LVAWs) and end-diastolic thickness (LVAWd), left ventricular ejection fraction (EF), and left ventricular fraction shortening (FS) were analyzed by Vevo LAB 5.6.0 depended on the mean values of three independent cardiac cycles of long-axis M-mode views.

**ELISA**

Before collecting peripheral blood, all mice were euthanized by an overdose of pentobarbital sodium (200 mg/kg). The plasma was centrifuged at 4℃ for 20 minutes (3000rpm) after anticoagulation for 10 minutes, and the supernatant was collected. The level of plasma CK-MB was quantified using a content assay kit (Nanjing Jiancheng, China) according to the manufacturer's instructions. The OD values were measured by microcoder at 450nm for analysis.

**Evans Blue/TTC and TUNEL**

To evaluate the ischemic and infarcted area, double-staining using triphenyltetrazolium

chloride (TTC) and Evans blue dye was performed. After completion of the preliminary protocol, 1% Evans blue was perfused to outline the risk area, and then the left ventricle was removed and cut into 1 mm slices. To quantify the size of the left ventricular scar, the left ventricular sections were incubated in 2% triphenyl tetrazole ammonium chloride (TTC). Non-ischemic area is stained blue, the area at risk (AAR) is stained red, while the infarct size (IS) is white. Three blind examiners quantified total myocardial area, risk area, and infarct area using digital planar measurements.

TUNEL staining (Roche, 11684817910) was performed using 4 μm thick sections of the peri-infarct zone fixed in ice-cold acetone/phosphate-buffered saline for 20 minutes. The slides were labeled in 37°C darkness for 60 min, permeated with 0.1% Triton X-100 and 5% Bovine Serum Albumin. Slides were rinsed with phosphate-buffered brine and nuclei were labeled with DAPI (Beyotime, Beijing, China). Images were obtained using a Laser confocal microscope. Tunel-positive cells were counted blinded to the experimental group and expressed as a percentage of all nuclei.

**Transmission electron microscopy (TEM) of cardiac tissue**

LV tissue samples were quickly excised, washed and fixed with 3% glutaraldehyde in phosphate buffer, embedded in epoxy resin, and then sectioned to 70–90 nm thickness. Electron micrographs were acquired and analyzed by TEM (Hitachi, Japan) for cardiac mitochondrial injury, including swelling and integrity.

**Cytoskeletal F-actin staining**

Phalloidin staining was performed using the peri-infarct frozen sections of heart tissue fixed in ice-cold acetone for 15 minutes. After cleaned by PBS twice, sections were stained by 5ug/ml TRITC-Phalloidin dye (Yeasen, Shanghai, China) at room temperature for 2 h, and the nucleus was stained by DAPI for 3 min. The excess water was absorbed, fluorescent sealing solution was added, and the cover glass was covered for fluorescent confocal microscope observation. To compare the relative quantity of actin polymerization, the average fluorescent intensity preimage was measured.

**RhoA activation assay**

RhoA activity assay was performed using pull-down assays (Cytoskeleton, BK036). The lysates were clarified by centrifugation at 16,000 g for 15 min at 4 °C. Supernatants were collected and incubated with Rhotenkin-Rho binding domain (RBD) Beads -and the mixture was rotated for 1h. The samples were washed with 500ul lysis/binding/wash buffer once and resuspended in 20ul 2x Laemmli sample buffer (125mM Tris pH 6.8, 20% glycerol, 4% SDS, 0.005% Bromophenol blue, 5% beta-mercaptoethanol), and the suspension was heated for 5 min at 95°C. Proteins were then isolated on 12.5% SDS-PAGE and detected by western blot. The activation of RhoA was determined by the protein level of GTP-RhoA.

**Cell culture and I/R injury treatment**

AC16 human cardiomyocyte-like cells were a kind gift from the College of Pharmacy at Harbin Medical University. H9C2 cell lines were purchased from BeNa Culture Collection (Beijing, China). AC16 cells and H9C2 cells were maintained in Dulbecco’s modified Eagle’s medium (DMEM, 12800-058, Gibco) supplemented with 10% FBS in a humidified atmosphere of 5% CO_2_ and 95% O_2_ at 37°C. The HUVECs were purchase from Chinese Academy of Science (Shanghai, China) and cultured in an F-12 1:1 61 (HyClone, Chicago, IL, USA) medium containing 10% FBS at 37 °C. Cells were used between the third and sixth passage.

For I/R treatment, the medium was replaced with warm serum and glucose-free DMEM prior to culture in a sealed hypoxic chamber (Billups-Rothenberg Modular Incubator Chamber, California, USA) at 37°C in an atmosphere of 5% CO_2_ and 95% N_2_ during the 6 h hypoxic stress period. For reoxygenation, the cellular medium was replaced with culture medium containing 1% FBS, and the cells were incubated under normoxic incubator conditions for 2 h.

**Neonatal mouse cardiomyocytes (NMCMs) culture**

Neonatal mouse cardiomyocytes (NMCMs) were isolated and cultured as previously described.^1^ Briefly, the hearts of 1-day-old C57BL/6J mice provided by the Laboratory Animal Center, the Second Affiliated Hospital of Harbin Medical University were quickly excised. CMs and cardiac fibroblasts were dispersed by digesting the hearts with 0.1% trypsin (27250-018, Gibco, Carlsbad, CA, USA) and 0.01% collagenase II (17101-015, Gibco) at 37 °C. The isolated cells were plated in 10-cm dishes for 1.5 h to allow the cardiac fibroblasts to attach. The unattached CMs were then transferred to new culture dishes for subsequent treatment. CMs were cultured in DMEM (12800-058, Gibco) containing 10% fetal bovine serum and antibiotics (15140-122, Gibco) at 37 °C in 95% O2 and 5% CO2.

**Preparation of small interfering RNA (si-RNA) and Transfection**

KIF5B small interfering RNAs were synthesized (GenePharma and RiboBio, China). The si-KIF5B primer sequences were as follows:

Rat: 5′-ACGUGACACGUUCGGAGAATT-3′

Human: 5′-AAACCGAGTTCCCTATGTAAA-3′

For si-KIF5B transfection, cells were seeded and grown to 60–70% confluency. After LIPUS treatment, the cells were added with OptiMEM contained Lipo 3000 and si-KIF5B. The transfection mix was replaced with fresh medium after 6h and analyzed post another 12h.

**Isolation of migrasomes**

Migrasomes were isolated using the Optiprep kit (Sigma-Aldrich, LY-SISO1) as previously described.^2^ In simple terms, cells were grown in DMEM petri dishes pre-coated with fibronectin (1mg/ml) for 12h. The cells were rinsed and trypsinized and collected in 50ml tubes. Centrifuge 1000g for 10 minutes, 4000g for 20 minutes to remove cells and large debris, and 18000g for 30 minutes all at 4 °C. The separation was then performed by iodixanol-sucrose density gradient centrifugation. The coarse particle samples were first suspended by mixing 400 ml extraction buffer with 400 ml 10% Optiprep. Build a ladder gradient from 50% (500 ml), then 40% (500 ml), 35% (500 ml), 30% (500 ml), 25% (500 ml), 20% (500 ml), 15% (500 ml), 10% (500 ml), 5% (500 ml) and crude migrasomes (5%, 800ml). Then, the prepared gradient liquid was centrifuged on the MLS-50 (Beckman) rotor at 150000 g for 4h. Finally, samples were collected from top to bottom, with 500 ml per distillate mixed with the same volume of PBS and centrifuged at 18000 g for 30 minutes to collect particles. The particles were washed with PBS and centrifuged again for 18000 g for 30 min. The samples were subjected to western blot analysis.

**Western blotting**

Total protein samples were extracted from heart tissues or cells lysed with RIPA Lysis Buffer (Beyotime, Shanghai, China). The protein was separated by 12.5% SDS-PAGE and electrically transferred to a polyvinylidene fluoride membrane followed by incubation with primary antibodies against TSPAN4 (1:1000, Novus), NDST1(1:1000, ABclonal), PIGK (1:1000, Abcam), EOGT (1:1000, Abcam), TOM20 (1:1000, Abcam), TIM23 (1:1000, Abcam), HSP60 (1:500, Wanleibio), MLC (1:1000, Cell Signaling Technology), PMLC (1:1000, Cell Signaling Technology), F-actin (1:1000, Abcam), YAP (1:1000, Cell Signaling Technology) and KIF5B (1:1000, Abcam) at 4℃ overnight and horseradish peroxidase-conjugated secondary antibodies for 1 h. Anti-β-actin (1:5000, Proteintech) and H3 antibodies (1:1000, Abcam) served as internal controls to normalize.

**Immunofluorescence confocal microscopy**

Cells grown on glass coverslips in 24-well culture plates were washed with PBS twice and fixed with 4% paraformaldehyde/PBS for 15 min. After permeabilized with 0.1% Triton X-100 for 30 min and blocked with 1% bovine serum albumin for 30 min, cells were incubated with anti-Tubulin (1:200, AC012, ABclonal), KIF5B (1:400, ab167429, Abcam) and Drp1 (1:400, ab184247, Abcam) overnight at 4°C and cells were stainedby TRITC-Phalloidin dye (Yeasen, Shanghai, China) for 30 minutes.

**Polymerase chain reaction (PCR)**

In cell-based real-time PCR experiments, total RNA was extracted directly with Trizol (Thermo Fisher Scientific, Waltham, Massachusetts), and using appropriate primers (Supplement Table1), RNA was converted to cDNA with a Transcriptor First Strand cDNA Synthesis Kit (Roche Diagnostics, Risch-Rotkreuz, Switzerland). Quantitative real-time PCR was performed using Universal SYBR qPCR Master Mix (Roche Diagnostics, Risch-Rotkreuz, Switzerland). The relative mRNA levels of KIF5B, Drp1 and Myo19 were normalized to GAPDH levels and analyzed by the 2^-△△ct^ method.

For the identification of mouse genotypes, PCR was performed using mouse genomic DNA as a template, and gel electrophoresis was used to compare the sizes of the specific products of different genotypes, and the differences in electrophoretic bands were used to directly distinguish the different genotypes of mice. The primers are listed in the Supplement Table 2.

**Cell Counting Kit 8 (CCK8) and Live/Dead assay**

Cell viability was detected by CCK8 (Dojindo Molecular Technology, Kumamoto, Japan) and Live/Dead staining in accordance with protocols. For CCK8, all cells (2×10^3^/well) were respectively seeded on 96-well plates at 37°C with 5% CO_2_. Five replicate wells were performed for each group. 10 µl CCK8 was added to each well and incubated in darkness for 4h at room temperature and the absorbance (OD) value was measured at 450 nm with a microplate reader. For Live/Dead assay, all cells (4×10^4^/well) were respectively seeded on 24-well plates. After being gently washed 3 times with 1×Assay Buffer, cells were incubated in Calcein AM and EthD-1 dye liquor for 15 min at 37 ℃, and then imaged as quickly as possible with fluorescence microscope.

**Flow Cytometry (FACS) for cellular apoptosis analysis**

After being washed thrice, cells were treated with trypsin and centrifuged. Cells of each pellet were stained with FITC-Annexin V and Propidium iodide (PI) in darkness, and FACS was conducted within 5 mins. Finally, the apoptotic cells were obtained using a FACS Calibur system (BD Biosciences, Franklin Lakes, NJ, USA), and the data were analyzed using the FlowJo software (Tree Star Corp, Ashland, OR, USA).

**JC-1 and DCFH-DA staining**

JC-1 and DCFH-DA were used to determine mitochondrial membrane potential and ROS according to the manufacturer’s manual. Briefly, after treatment as described, cells were gently washed once with warm PBS. Cells were incubated in JC-1 or 2,7-dichlorodihydrofluorescein diacetate (DCFH-DA) working solution for 20 min at 37 ℃, and then gently washed three times with warm PBS for fluorescence microscopy assay or fluorescence microplate reader to determine the fluorescence intensity.

**Cellular oxygen consumption rate (OCR)**

OCR of cells was measured using Seahorse XFe96 extracellular flux analyzer (Seahorse Bioscience) according to manufacturer's instructions. In brief, 5000 cells (per well) were cultured for 12 h in FN-precoated XFe96 well plates containing the medium after treatment as described above. Prior to measurement, the cell medium was replaced by XF assay medium (XF base medium added 25 mM glucose, 1 mM pyruvate, 6 mM L-glutamine, and adjusted to pH 7.4). The cells were then placed in 175 mL/ well XF assay medium and stored in a CO_2_-free incubator at 37℃ for 1 h. During incubation, 16 mM oligomycin (8×), 18 mM FCCP (9×), 10 mM rotidone (10×) and 10 mM Mantemycin A (10×) in the XF test medium were loaded into the injection port of the XFe96 sensor cartridge, respectively. The determination procedure was set as "mixing -03:00, waiting -00:00, determination -03:00", a total of 4 cycles. After measurement, the cells were digested with trypsin and counted for cell number. Cell OCR was normalized by cell number. Wave software (Agilent Technologies) was used to analyze the data set.

**Transwell and scratch/wound assay**

Transwell and Scratch/Wound methods were applied to assess the capacity of migration.

Cells were inoculated with serum-free medium into transwell implant (Corning, USA) with 10% serum medium at the base of the implant. After 24 h, the cells were fixed with 4% paraformaldehyde and stained by 0.3% crystal violet.

For the scratch/wound assay, cells were cultured to 100% confluence in 6-well plates. 200 µL pipette tip was used to scratch the cell monolayer. The scratched cells were then cultured in serum-free medium for 24 hours. Olympus BX51 vertical microscope was adopted to reap the images. Migration rate = (wound area (0h) - wound area (24h)) / wound area (0h) )×100%. All assays were repeated three times.

**Cell transmission electron microscopy**

The AC16 cells and HUVEC samples were cultivated and obtained in 1.5 ml EP tubes, and centrifuged at 2000 rpm for 15 min to ensure that the cells were clumped together. The supernatant was removed, and the samples were washed and fixed with glutaraldehyde in phosphate buffer, embedded in epoxy resin, and then sectioned. Electron micrographs were acquired and analyzed by TEM (Hitachi, Japan) for migrasomes quantity and mitochondrial status in cells and migrasomes.

**RNA sequencing**

RNA screening of I/R AC16 cells with or without LIPUS irradiation was performed using the Illumina HiSeq 2500 platform (OE Biotechnology Co, LTD, Shanghai, China). Total RNA was extracted from the samples with Trizol and converted to cDNA. Then the cDNA structure was amplified by polymerase chain reaction (PCR). DESeq2 R packages was used for differential analysis and enrichment analysis (Gene ontology and Kyoto encyclopedia of genes and genomes). The identified differentially expressed genes (DEGs) must match selection criteria: p-value < 0.05 and fold change (FC) >1.5.

**Chromatin immunoprecipitation (CHIP)**

ChIP assay kit (Cell signaling Technology, USA) was used to prove the binding of transcription factor TEAD and DNA-sequences as described in manufacturer's instructions. After cell lysis, isolated nuclei were subjected to sonication for small chromatin fragments. Sheared chromatin was diluted and divided into aliquots for immunoprecipitation. The chromatin and the transcription factors interacting with it were cross-linked by 1% formaldehyde, then the TEAD-specific antibody was added. The antibody-transcription faction-chromatin complexes was dragged down using magnetic protein A/G magnetic beads, and the DNA sequence bound to TEAD was detected by qRT-PCR followed by purified and recovered using appropriate primers (Supplement Table 3).

## Supplemental Tables

### Supplement Table 1. The primer and sequence of qRT-PCR

| **Primer** | | **Forward 5’-3’** | **Reverse 5’-3’** |
| --- | --- | --- | --- |
| Kif5b  Drp1  Myo19  GAPDH | AATTGTGTCCTCTGTCGTTGTCTTC  AGGCAGGAGGATTCGCTTGAG  GCCTGGAGTGGTCATTCATCAAC  AGAAGGCTGGGGCTCATTTG | | GGCGGAGGTAGGTTACAGTGAG  GGACTACACGCATACACCATCAC  ACTCTGGTGCTCGGCTCATTAG  AGGGGCCATCCACAGTCTTC |

### Supplement Table 2. The primer and sequence of PCR for genotyping strategy

| **Primer** | | **Forward 5’-3’** | **Reverse 5’-3’** |
| --- | --- | --- | --- |
| Tspan4LoxP  αMHC-MerCreMer  Cdh5-CreERT2 | TTGATTATGAGCAGTAGTGTGCCA  GCGGTCTGGCAGTAAAAACTATC  TTGTGGATTTGACCCTCCATGAT | | TATGTAGGCCATGGCAATTGAGT  GTGAAACAGCATTGCTGTCACTT  CACGTTCTTGCACTTCATGCTG |

### Supplement Table 3. The primer and sequence of ChIP-PCR

| **Primer** | | **Forward 5’-3’** | **Reverse 5’-3’** |
| --- | --- | --- | --- |
| KIF5B  Drp1 | ACCAGTATTAGATTCATACTTCTGCA  TAGAGTTCTGCGGGTGTTGG | | AGGAGTGCTATTTTCTCACACCT  TCGAAGTAGCAGAGAAAAGGAAAAT |

## Supplemental Figures

**
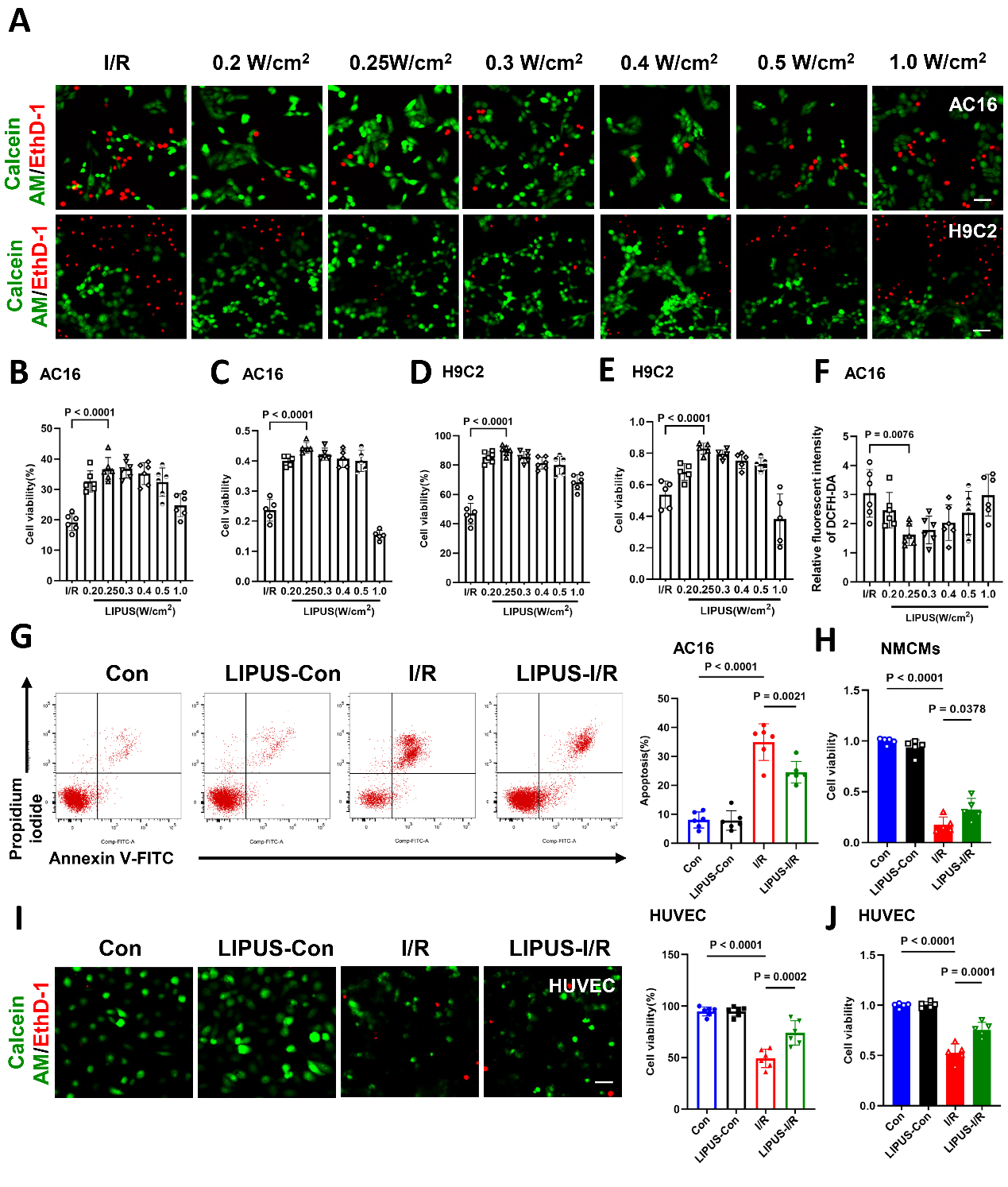
Supplemental Figure 1. Low-intensity pulsed ultrasound (LIPUS) therapy enhanced cell viability and reduced cell apoptosis post-I/R** **(A)** Fluorescence staining with calcein-AM (green) vital dyes represents living cells, and ethidium homodimer-1 staining (red) represents dead cells in AC16 and H9C2 cells. **(B)** The percentage of cell viability of AC16 cells was calculated. The cell viability of Live/dead assay with a maximum effect noted at 0.25 W/cm^2^ (n = 6). **(C)** The cell viability was measured with CCK8 kit in AC16 at varied pressure conditions (n = 5). **(D)** The percentage of cell viability of H9C2 cells was calculated (n = 6). **(E)** The cell viability was measured with CCK8 kit in H9C2 cells at varied pressure conditions (n = 5). **(F)** The relative fluorescent of DCFH-DA in AC16 cells was calculated. **(G)** Flow cytometry analysis of Annexin V-APC and PI fluorescence in AC16 cell lines post-I/R and LIPUS treatment with 0.25 W/cm^2^. The percentage of AC16 cell apoptosis is shown in right panels. (n = 6). **(H)** The cell viability measured by CCK8 in neonatal mouse cardiomyocytes (NMCMs) post-I/R and LIPUS treatment with 0.25 W/cm^2^ (n = 5). **(I)** Live/dead assay and the cell viability of HUVECs post-I/R and LIPUS treatment with 0.25 W/cm2 (n = 6). **(J)** The cell viability measured by CCK8 in HUVECs post-I/R and LIPUS treatment with 0.25 W/cm^2^ (n = 5). Scale bar, 50 μm in A-H. Results are expressed as mean ± SD. Comparisons of parameters were performed with ANOVA followed by Tukey’s test for multiple comparisons.

**
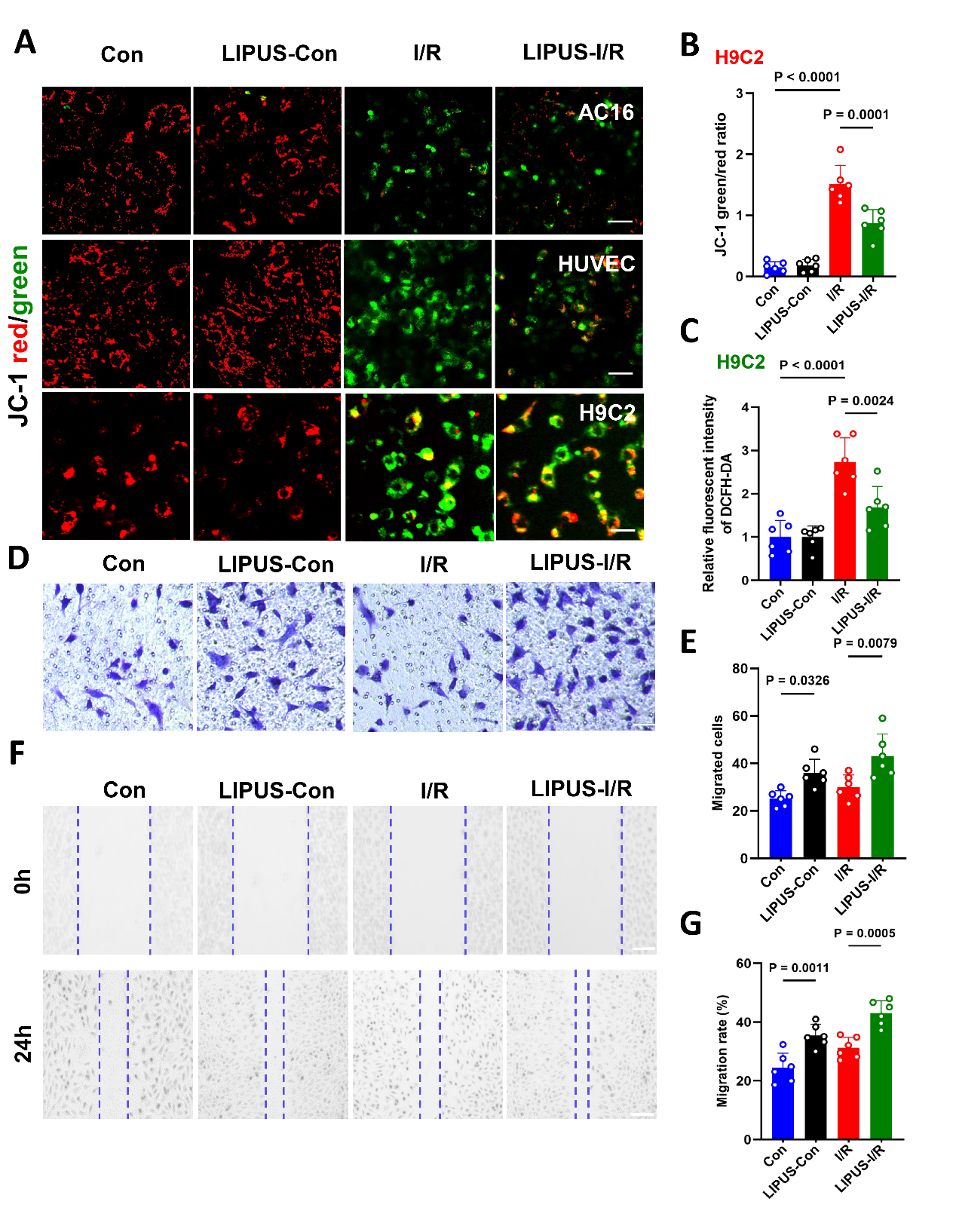
**

**Supplemental Figure 2. Low-intensity pulsed ultrasound (LIPUS) therapy improved mitochondrial homeostasis and promoted cell movement *in vitro* (A)** Representative JC-1 staining of AC16, H9C2 cells and HUVECs. Scale bar, 50 μm. **(B)** The percentage of JC-1 green/red ratio in H9C2 cells was calculated. **(C)** The relative fluorescent of DCFH-DA in H9C2 cells was calculated and shown. **(D, E)** Transwell assay was performed in HUVECs and the count of migrated cells was measured. Scale bar, 40 μm. **(F, G)** Scratch/wound assay was performed in HUVECs and the migration rate was calculated. Scale bar, 100 μm. (n = 6). Results are expressed as mean ± SD. Comparisons of parameters were performed with ANOVA followed by Tukey’s test for multiple comparisons.

**
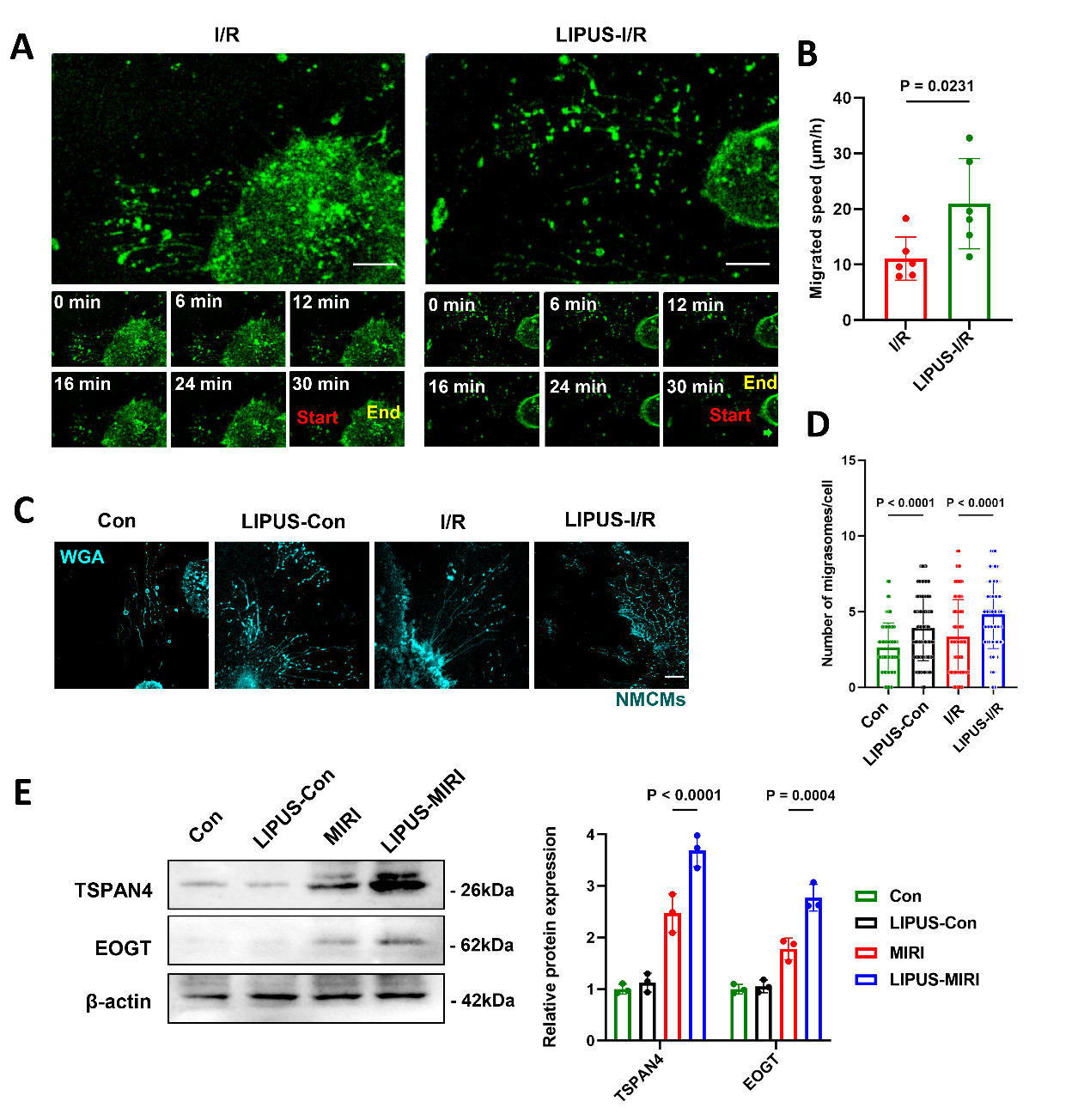
**

**Supplemental Figure 3. Low-intensity pulsed ultrasound (LIPUS) facilitated cell migration and migrasomes formation (A, B)** Living cells confocal image of AC16 cells expressing TSPAN4-GFP. Scale bar, 20 μm. The migrated speed of AC16 cells measured by living cells imaging (n = 6). **(C, D)** Confocal image of NMCMs post-I/R and LIPUS stimulation stained with WGA, and the number of migrasomes was calculated. n = 120. Scale bar, 20 μm. **(E)** Western blot analysis for the migrasomes markers of cardiac tissue (N = 3 independent repeats).

**
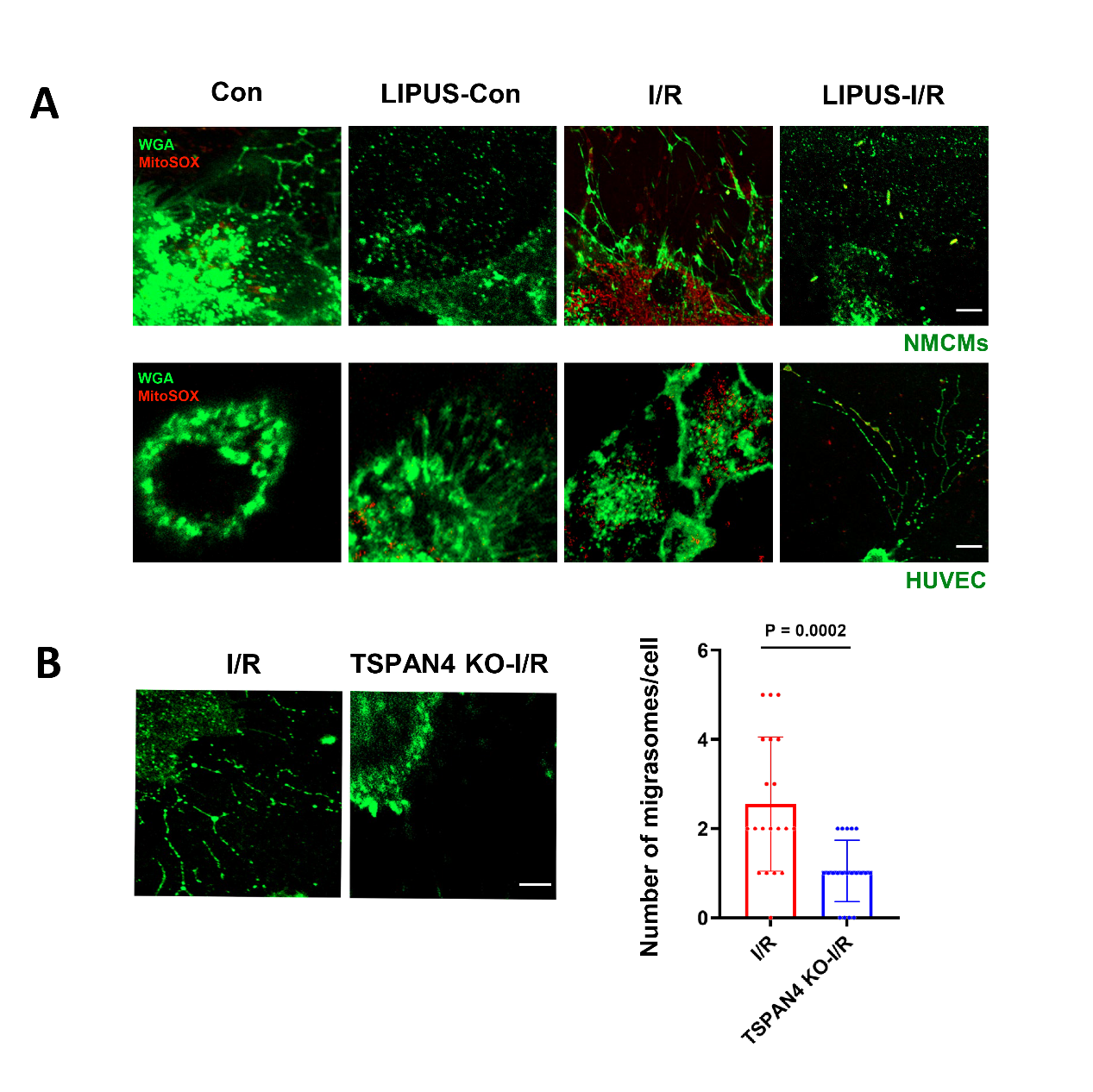
**

**Supplemental Figure 4. Low-intensity pulsed ultrasound (LIPUS) therapy promoted damaged mitochondria excretion via migrasomes of NMCMs and HUVECs (A)** Confocal image of NMCMs and HUVECs post-I/R and LIPUS stimulation stained with WGA and MitoSOX. Scale bar, 20 μm. **(B)** Representative confocal image of TSPAN4 KO on migrasome formation in HUVECs stained with WGA and the migrasomes number of each cell. n = 20. Scale bar, 20 μm.
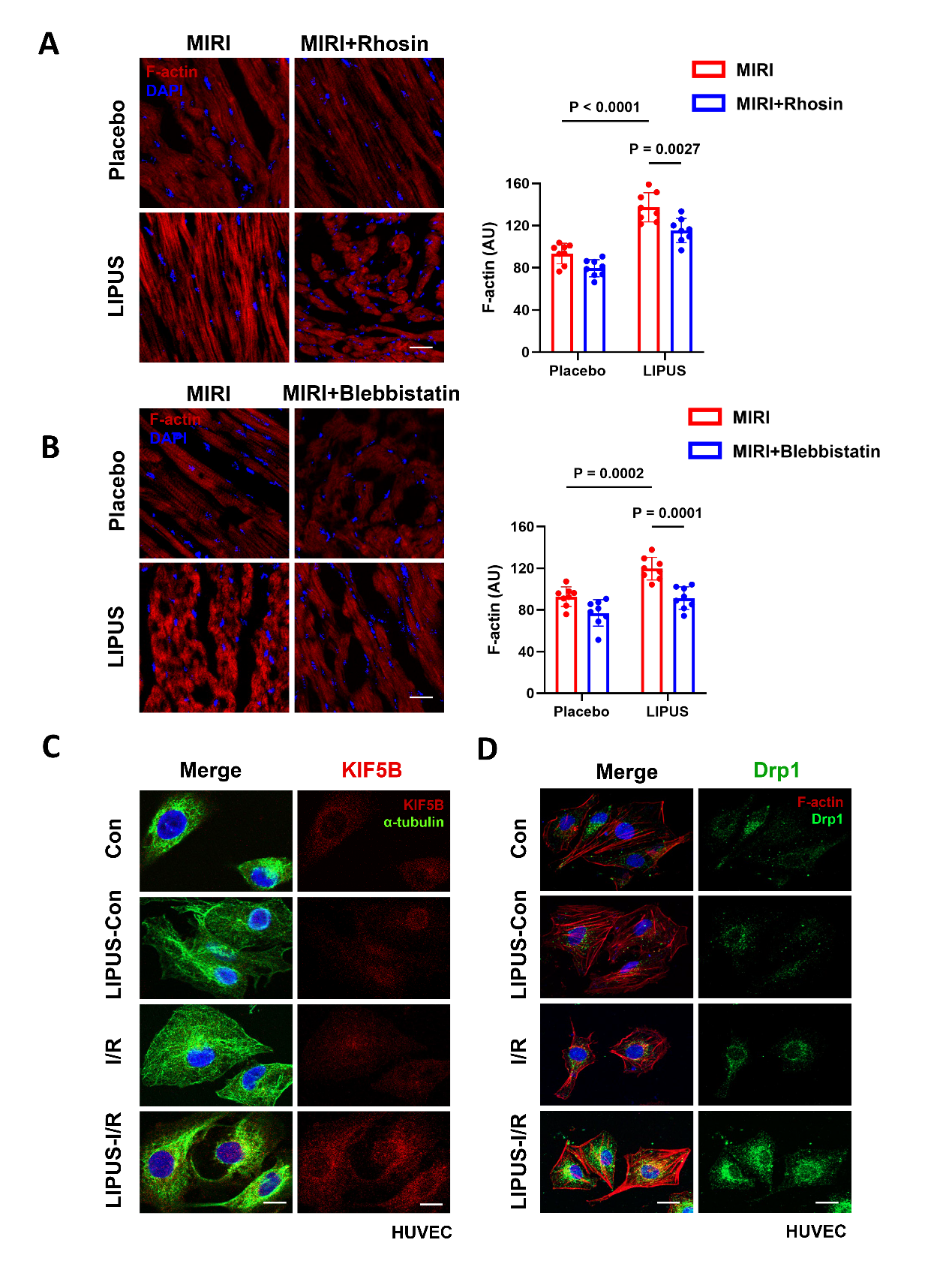


**Supplemental Figure 5. Inhibition of RhoA and myosin II decreased the expression of KIF5B and Drp1 induced by LIPUS (A)** Immunofluorescence staining with phalloidin (red) of cardiac tissue in mice treated with Rhosin and LIPUS. **(B)** Immunofluorescence staining with phalloidin (red) of cardiac tissue in mice treated with Blebbistatin and LIPUS. Scale bar, 100 µm, n = 8 in A and B. **(C)** Immunofluorescence co-staining with KIF5B (red) and microtublin (green) in HUVECs post-I/R and LIPUS treatment. **(D)** Immunofluorescence staining with Drp1 (green) and F-actin (red) in HUVECs post-I/R and LIPUS treatment. Scale bar, 50 µm in C and D. Results are expressed as mean ± SD. Comparisons of parameters were performed with ANOVA followed by Tukey’s test for multiple comparisons.


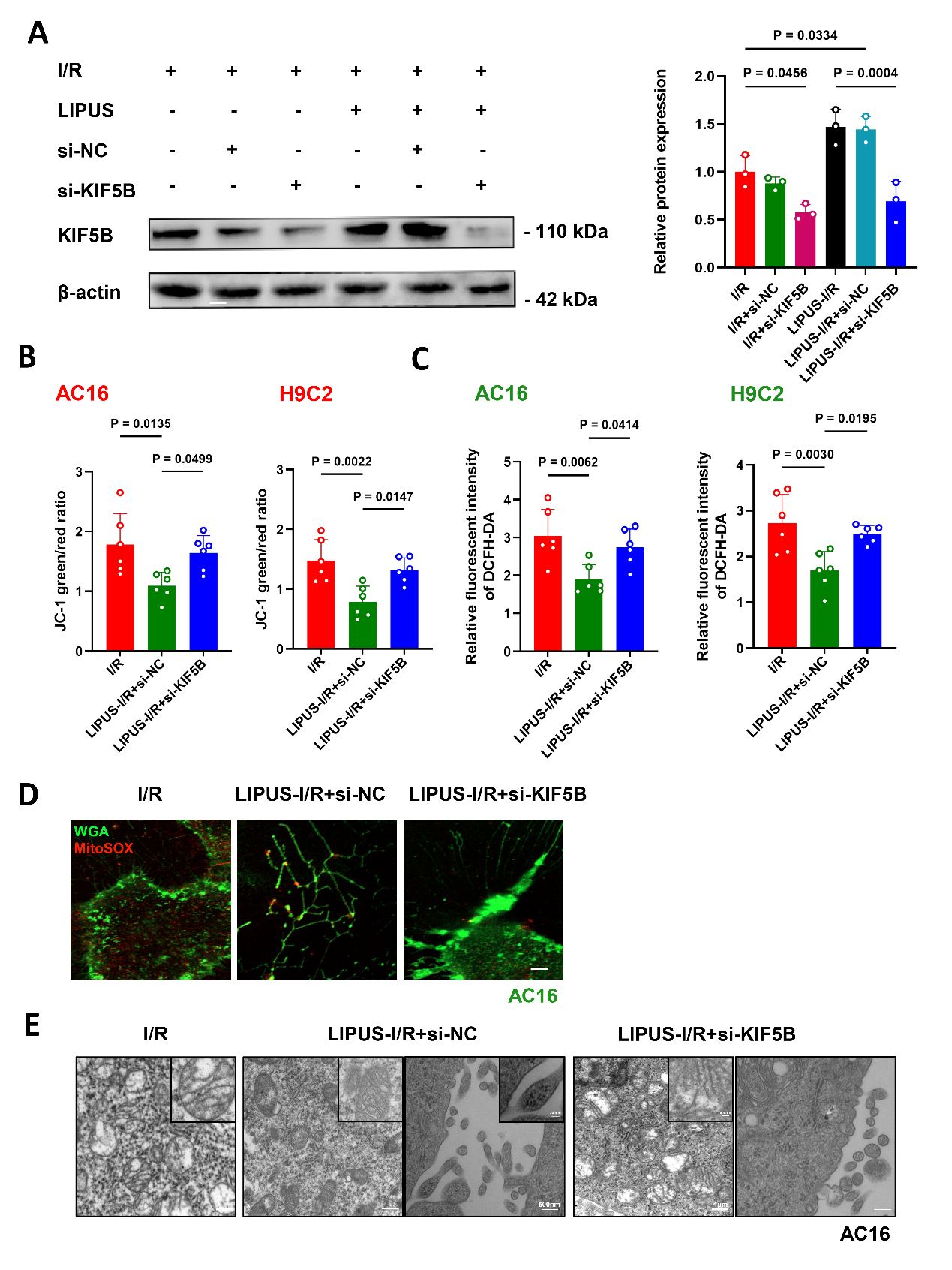


**Supplemental Figure 6. Absence of the beneficial effects of low-intensity pulsed ultrasound (LIPUS) on ischemia-reperfusion injury (I/R) in si-KIF5B cells (A)** Western blot analysis of AC16 cells with or without si-KIF5B transfection (N = 3 independent repeats). **(B)** The percentage of JC-1 green/red ratio in H9C2 and AC16 transfected with si-KIF5B and treated with LIPUS was calculated. **(C)** The relative fluorescent intensity of DCFH-DA in H9C2 and AC16 cells transfected with si-KIF5B and treated with LIPUS was measured (n = 6). **(D)** Confocal image of AC16 cells transfected with si-KIF5B and treated with LIPUS stained with WGA and MitoSOX. Scale bar, 20 μm. **(E)** Representative TEM images of AC16 cells transfected with si-KIF5B and treated with LIPUS. Scale bar, 1 μm. Enlarged ROI. Scale bar, 500 nm. Enlarged ROI. Scale bar, 200 nm. Results are expressed as mean ± SD. Comparisons of parameters were performed with ANOVA followed by Tukey’s test for multiple comparisons.


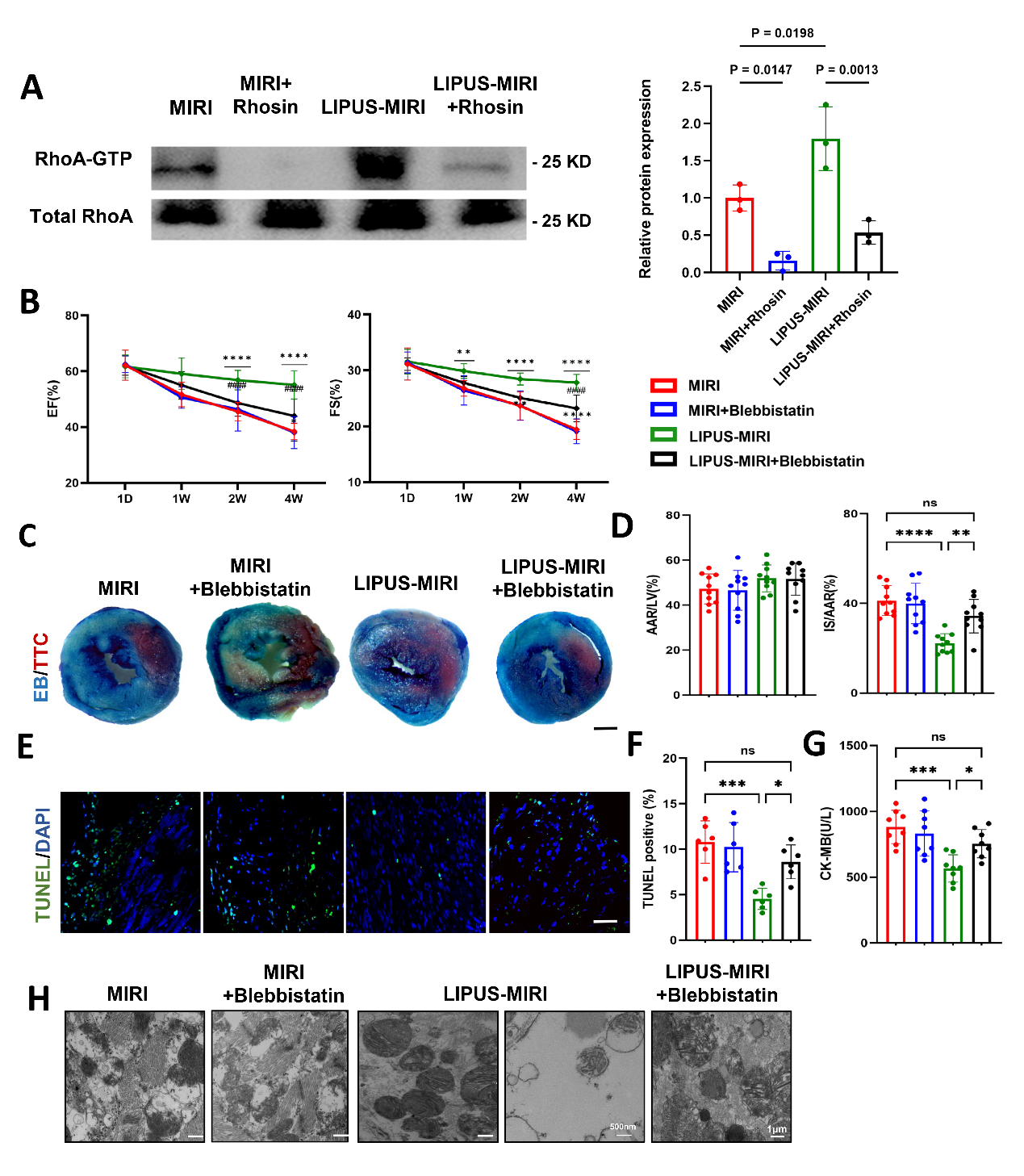


**Supplemental Figure 7. Absence of the beneficial effects of low-intensity pulsed ultrasound (LIPUS) on MIRI improvement and damaged mitochondria discharge in mice with myosin II inhibition** **(A)** Western blot analysis of cardiac tissue with or without Rhosin (N = 3 independent repeats). **(B)** Echocardiogram measurements in MIRI mice treated with Blebbistatin and LIPUS (LVEF: 1W: ^*^P = 0.0070; 2W: ^*^P < 0.0001; ^#^P= 0.0023; 4W: ^*^P < 0.0001; ^*^P = 0.0459; ^#^P < 0.0001; LVFS: 1W: ^*^P = 0.0032; 2W: ^*^P < 0.0001; ^#^P = 0.0010; 4W: ^*^P < 0.0001; ^*^P < 0.0001; ^#^P < 0.0001; LVAWs: P = 0.0008 ^*^MIRI group vs LIPUS-MIRI group; P = 0.0303 ^*^LIPUS-MIRI group vs LIPUS-MIRI+Blebbistatin group; LVAWd: P = 0.0113 ^*^MIRI group vs LIPUS-MIRI group; P = 0.0420 ^*^LIPUS-MIRI group vs LIPUS-MIRI+Blebbistatin group, n = 10). **(C, D)** Representative images of Evans blue/TTC staining and quantification of AAR and IS in MIRI mice treated with Blebbistatin and LIPUS (scale bar, 1 mm, P < 0.0001 ^*^MIRI group vs LIPUS-MIRI group; P = 0.0027 ^*^LIPUS-MIRI group vs LIPUS-MIRI+Blebbistatin group, n = 10). **(E, F)** TUNEL immunofluorescence staining identifies apoptotic cells, followed by DAPI staining (scale bar, 100 μm, P = 0.0003 ^*^MIRI group vs LIPUS-MIRI group; P = 0.0149 ^*^LIPUS-MIRI group vs LIPUS-MIRI+Blebbistatin group, n = 6). **(G)** Serum CK-MB levels at 3 days after MIRI onset treated with Blebbistatin and LIPUS. **(H)** Representative TEM images of cardiac tissue in indicated group. Scale bar, 1 μm (P = 0.0002 ^*^MIRI group vs LIPUS-MIRI group; P = 0.0359 ^*^LIPUS-MIRI group vs LIPUS-MIRI+Blebbistatin group, n = 8). Results are expressed as mean ± SD. Comparisons of parameters were performed with ANOVA followed by Tukey’s test for multiple comparisons; (^*^P < 0.05; ^**^P < 0.01; ^***^P < 0.001; ^****^P < 0.0001; ^#^P < 0.05; ^##^P < 0.01; ^##^^##^P < 0.0001; ^*^ MIRI-no LIPUS group vs MIRI-LIPUS group; ^*^MIRI+Blebbistatin-no LIPUS group vs MIRI+Blebbistatin-LIPUS group; P < 0.0001^#^MIRI-LIPUS group vs MIRI +Blebbistatin-LIPUS group).

**
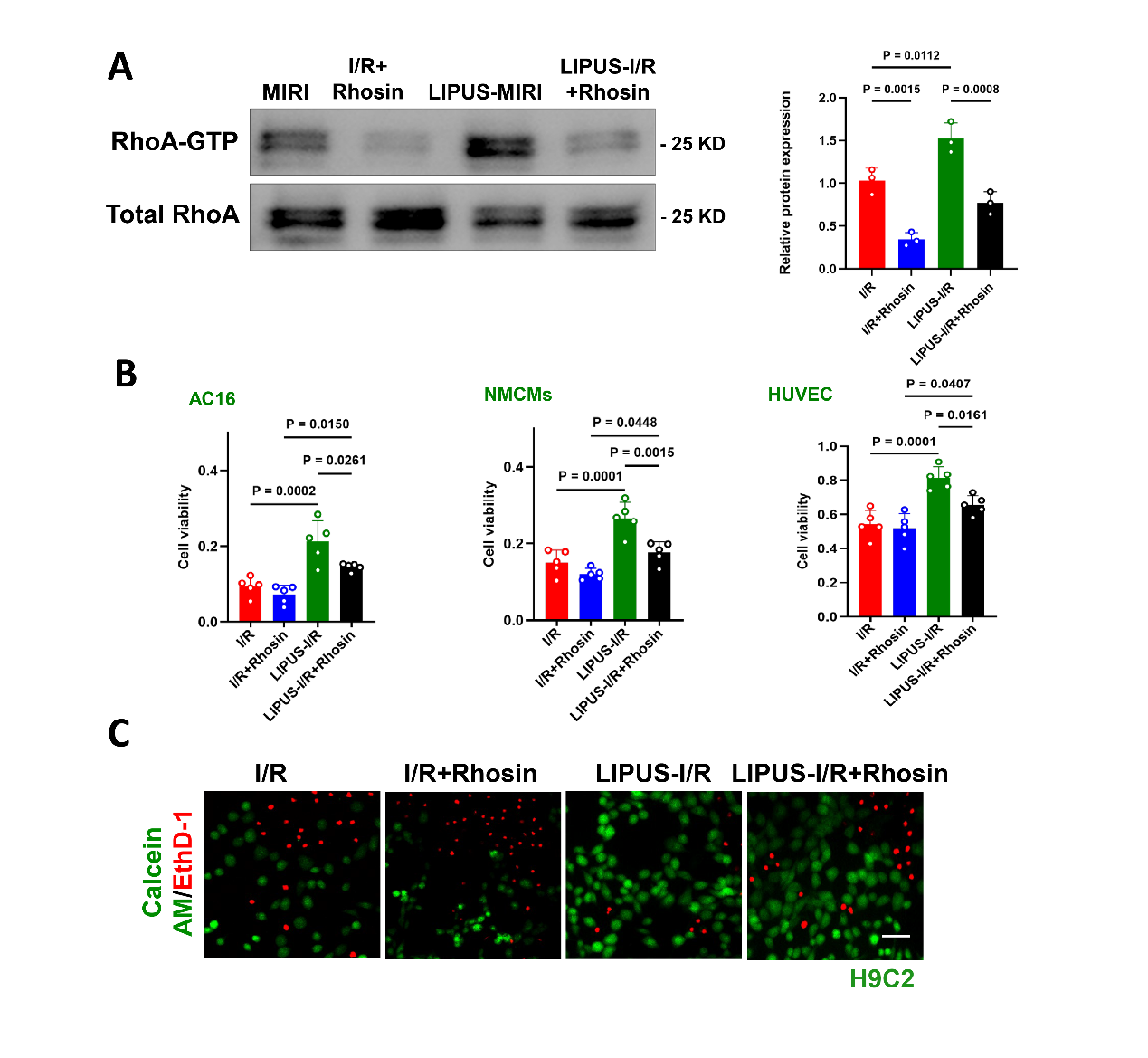
**

**Supplemental Figure 8. Absence of the cardiomyocyte protection post-I/R of low-intensity pulsed ultrasound (LIPUS) in cells treated with Rhosin** **(A)** Western blot analysis of indicated AC16 cells with or without Rhosin (N = 3 independent repeats). **(B)** The cell viability of CCK8 in AC16 cells, NMCMs and HUVECs (n = 5). **(C)** Live/dead assay of H9C2 treated with Rhosin and LIPUS. Results are expressed as mean ± SD. Comparisons of parameters were performed with ANOVA followed by Tukey’s test for multiple comparisons.


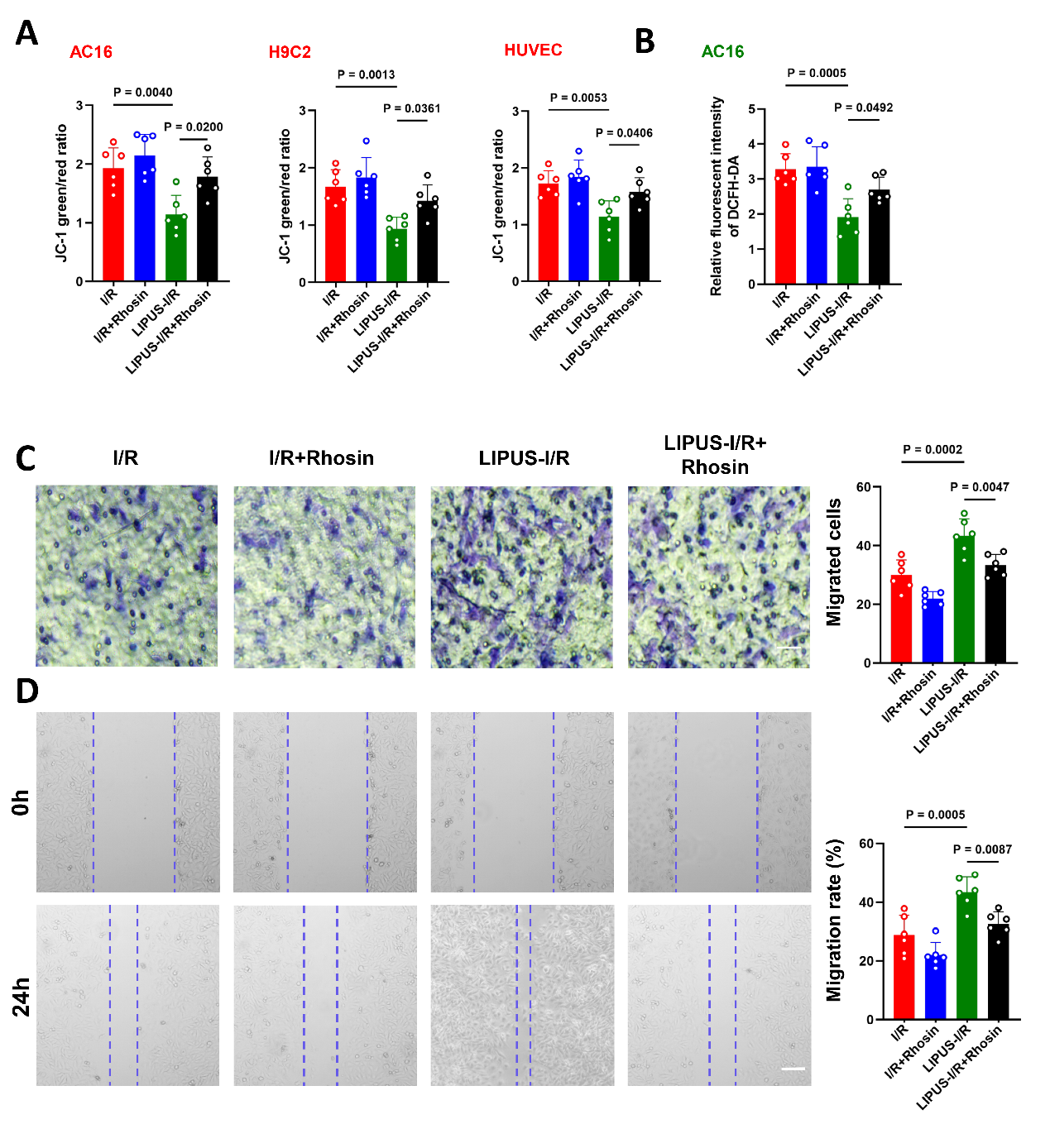


**Supplemental Figure 9.** **Absence of the improved mitochondrial homeostasis and promoted cell movement of low-intensity pulsed ultrasound (LIPUS) in cells treated with Rhosin post-I/R in vitro (A)** The percentage of JC-1 green/red ratio of AC16, H9C2, HUVEC cells treated with Rhosin and LIPUS were shown. Scale bar, 50 μm (n = 6). **(B)** The relative fluorescent intensity of DCFH-DA in AC16 cells treated with Rhosin and LIPUS (n = 6). **(C)** Transwell assay was performed in HUVECs treated with Rhosin and LIPUS and the count of migrated cells was measured. Scale bar, 40 μm. **(D)** Scratch/wound assay was performed in HUVECs treated with Rhosin and LIPUS and the migration rate was calculated. Scale bar, 100 μm. (n = 6). Results are expressed as mean ± SD. Comparisons of parameters were performed with ANOVA followed by Tukey’s test for multiple comparisons.

**
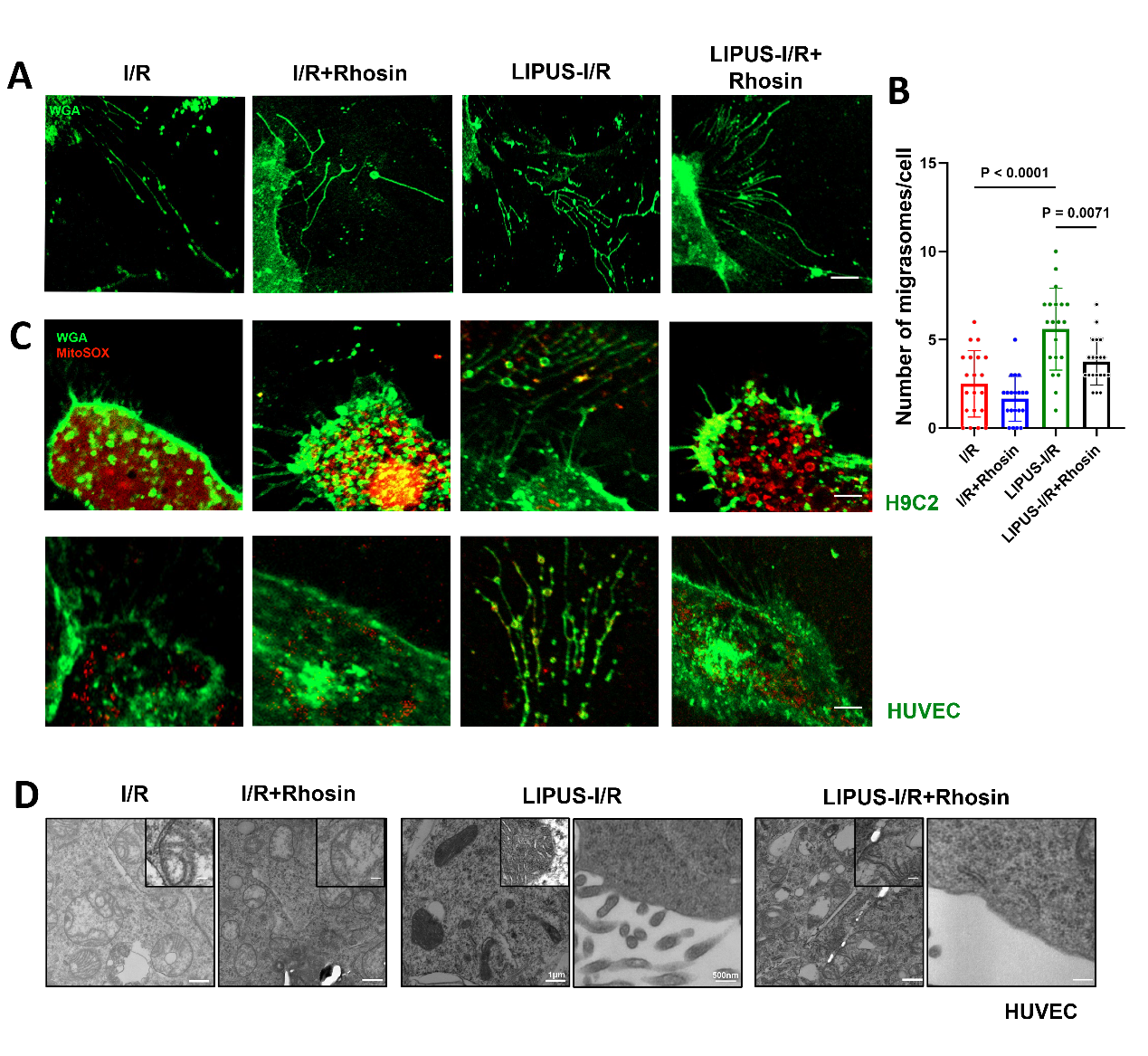
**

**Supplemental Figure 10.** **Absence of the mitocytosis of low-intensity pulsed ultrasound (LIPUS) in cells treated with Rhosin post-I/R** **(A, B)** Representative confocal image of HUVECs treated with Rhosin and LIPUS followed by stained with WGA and the migrasomes number of each cell. n = 20. Scale bar, 20 μm. **(C)** Confocal image of H9C2 cells and HUVECs treated with Rhosin and LIPUS followed by stained with WGA and MitoSOX. Scale bar, 20 μm. **(D)** Representative TEM images of HUVECs treated with Rhosin and LIPUS. Scale bar, 1 μm. Enlarged ROI. Scale bar, 500 nm. Enlarged ROI. Scale bar, 200 nm. Results are expressed as mean ± SD. Comparisons of parameters were performed with ANOVA followed by Tukey’s test for multiple comparisons.


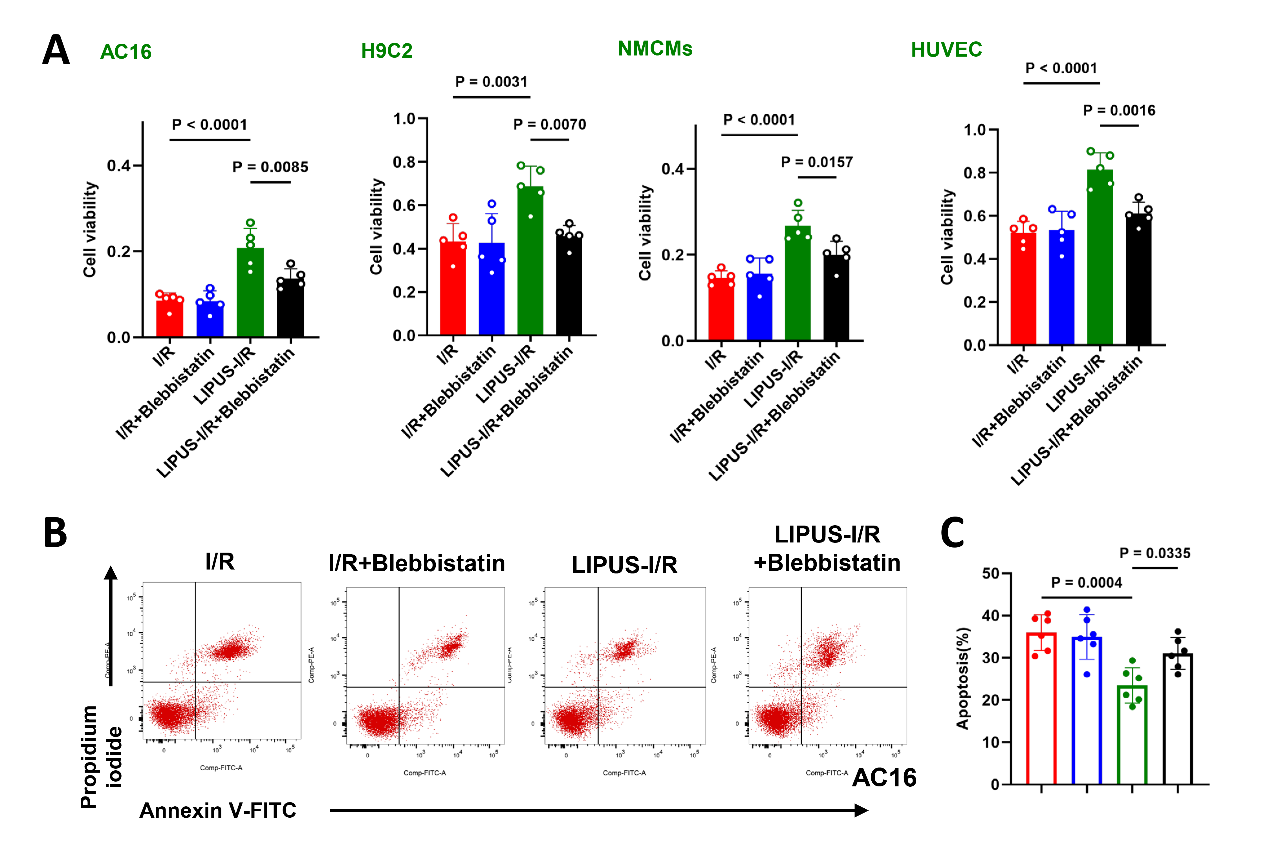


**Supplemental Figure 11.** **Absence of the cardiomyocyte protection of low-intensity pulsed ultrasound (LIPUS) in cells treated with Blebbistatin post-I/R** **(A)** The cell viability of CCK8 in H9C2 and AC16 cells, NMCMs and HUVECs treated with Blebbistatin and LIPUS (n = 5). **(B)** Representative flow cytometry results of Annexin V-APC and PI fluorescence in AC16 cell lines. (**C**) The percentage of cell apoptosis of flow cytometry analysis in AC16 cells and quantification of abnormal mitochondria in AC16 cells treated with Blebbistatin and LIPUS (n = 6). Comparisons of parameters were performed with ANOVA followed by Tukey’s test for multiple comparisons.


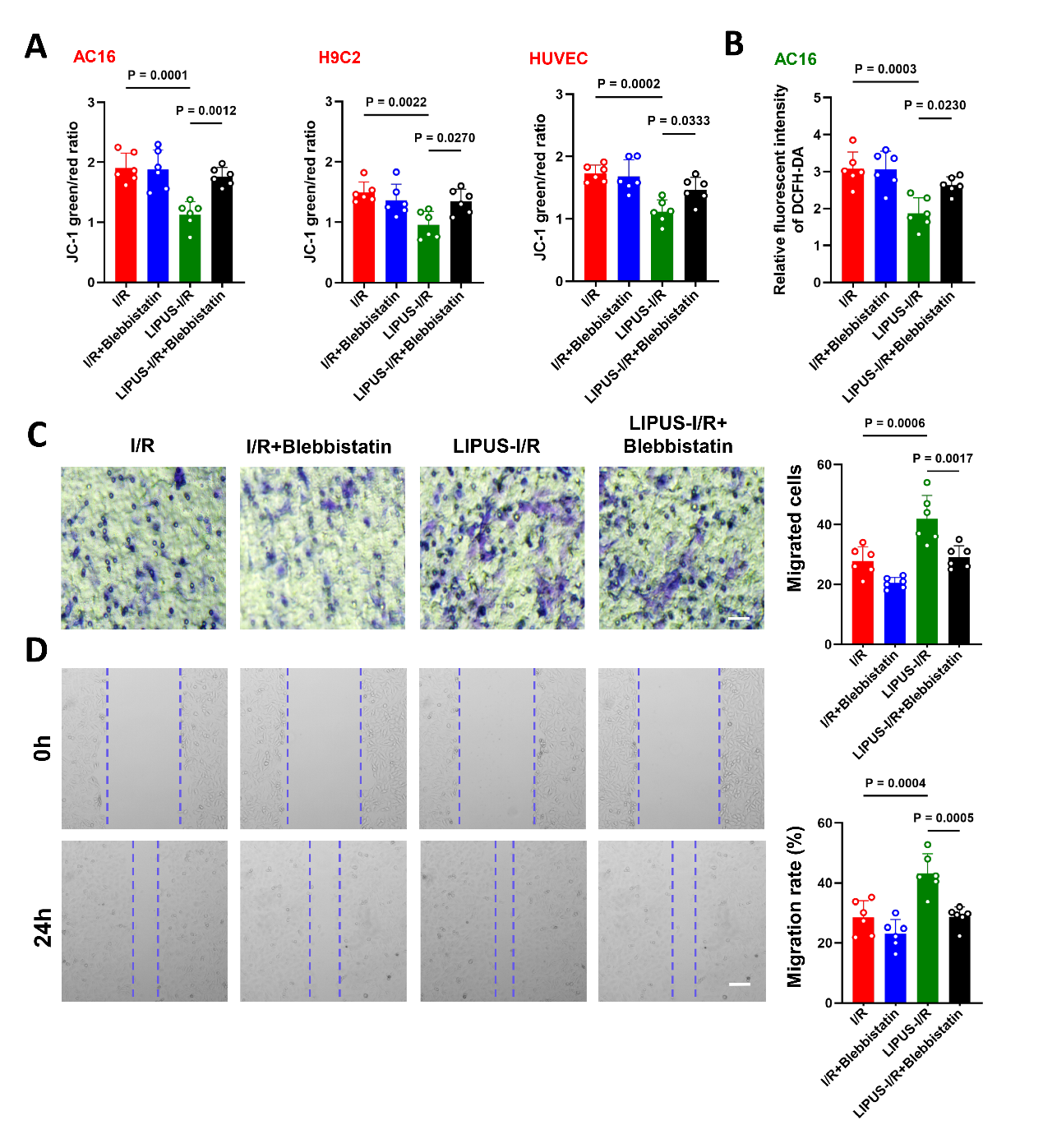


**Supplemental Figure 12. Absence of the improved mitochondrial homeostasis and promoted cell movement of low-intensity pulsed ultrasound (LIPUS) in cells treated with Blebbistatin post-I/R in vitro (A)** JC-1 staining of AC16, H9C2, HUVEC cells treated with Blebbistatin and LIPUS. The percentage of JC-1 green/red ratio was calculated (n = 6). **(B)** The relative fluorescent intensity of DCFH-DA in AC16 cells treated with Blebbistatin and LIPUS (n = 6). **(C)** Transwell assay was performed in HUVECs treated with Blebbistatin and LIPUS and the count of migrated cells was measured. Scale bar, 40 μm. **(D)** Scratch/wound assay was performed in HUVECs treated with Blebbistatin and LIPUS and the migration rate was calculated. Scale bar, 100 μm. (n = 6). Results are expressed as mean ± SD. Comparisons of parameters were performed with ANOVA followed by Tukey’s test for multiple comparisons.


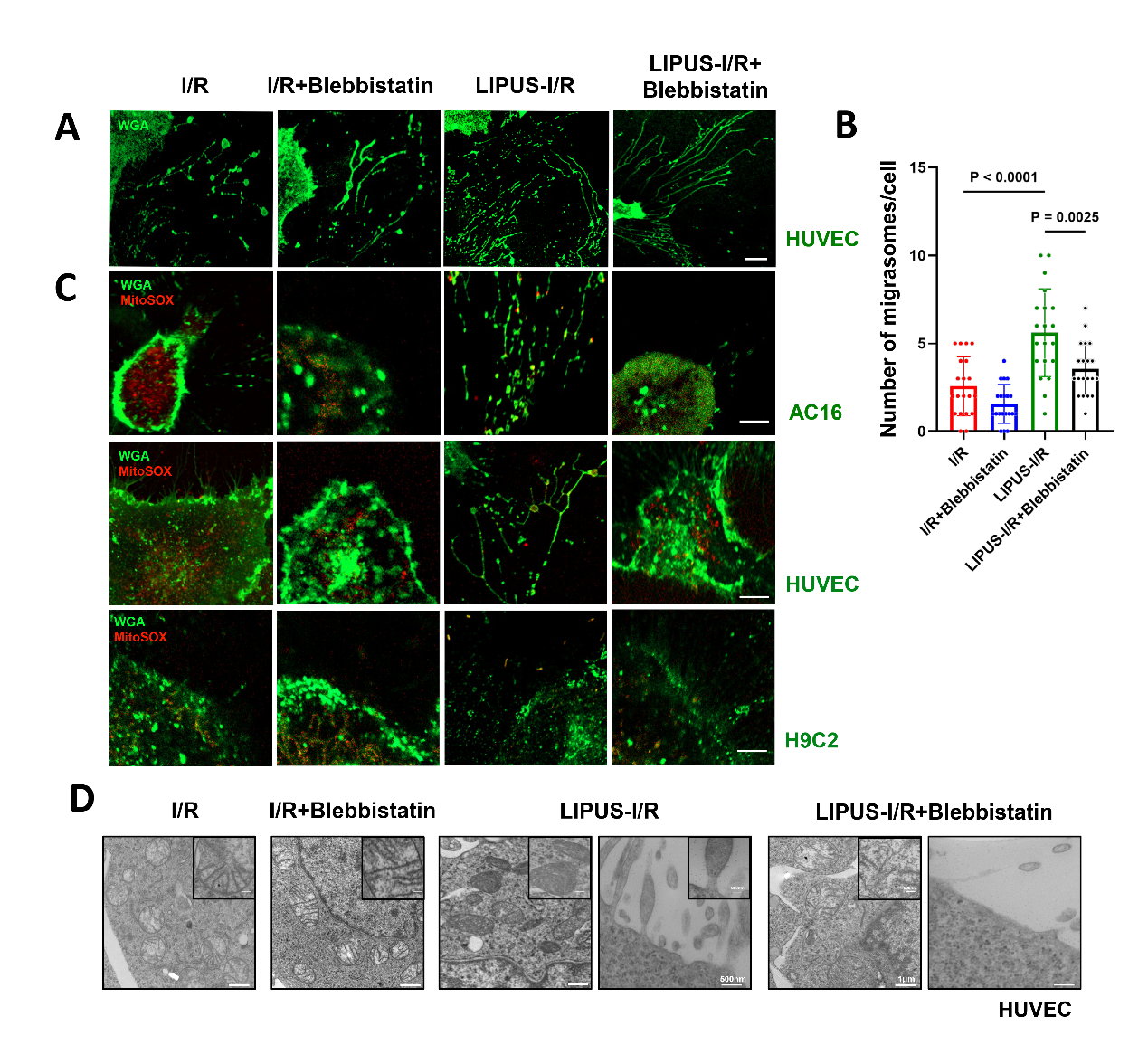


**Supplemental Figure 13. Decreased mitocytosis of low-intensity pulsed ultrasound (LIPUS) in cells treated with Blebbistatin post I/R (A, B)** Representative confocal image of HUVECs treated with Blebbistatin and LIPUS followed by stained with WGA, and the number of migrasomes was calculated. n = 20. Scale bar, 20 μm. **(C)** Confocal image of AC16, H9C2 cells and HUVECs treated with Blebbistatin and LIPUS followed by stained with WGA and MitoSOX. Scale bar, 20 μm. **(D)** Representative TEM images of HUVECs cells treated with Blebbistatin and LIPUS. Scale bar, 1 μm. Enlarged ROI. Scale bar, 500 nm. Enlarged ROI. Scale bar, 200 nm. Results are expressed as mean ± SD. Comparisons of parameters were performed with ANOVA followed by Tukey’s test for multiple comparisons.

**References**

1. Simon JN, Vrellaku B, Monterisi S, et al. Oxidation of Protein Kinase A Regulatory Subunit PKARIalpha Protects Against Myocardial Ischemia-Reperfusion Injury by Inhibiting Lysosomal-Triggered Calcium Release. *Circulation.* 2021;143(5):449-465.

2. Jiao H, Jiang D, Hu X, et al. Mitocytosis, a migrasome-mediated mitochondrial quality-control process. *Cell.* 2021;184(11):2896-2910 e2813.
